# Aboveground and belowground biodiversity have complementary effects on ecosystem functions across global grasslands

**DOI:** 10.1371/journal.pbio.3002736

**Published:** 2024-08-14

**Authors:** Catarina S. C. Martins, Manuel Delgado-Baquerizo, Ramesha H. Jayaramaiah, Dongxue Tao, Jun-Tao Wang, Tadeo Sáez-Sandino, Hongwei Liu, Fernando T. Maestre, Peter B. Reich, Brajesh K. Singh

**Affiliations:** 1 Hawkesbury Institute for the Environment, Western Sydney University, Penrith, Australia; 2 Laboratorio de Biodiversidad y Funcionamiento Ecosistémico, Instituto de Recursos Naturales y Agrobiología de Sevilla (IRNAS), CSIC, Sevilla, Spain; 3 Institute of Grassland Science, Key Laboratory of Vegetation Ecology of the Ministry of Education, Jilin Songnen Grassland Ecosystem National Observation and Research Station, Northeast Normal University, Changchun, China; 4 Environmental Sciences and Engineering, Biological and Environmental Science and Engineering Division, King Abdullah University of Science and Technology, Thuwal, Kingdom of Saudi Arabia; 5 Instituto Multidisciplinar para el Estudio del Medio “Ramón Margalef”, Universidad de Alicante, San Vicente del Raspeig, Alicante, Spain; 6 Department of Forest Resources, University of Minnesota, Saint Paul, Minnesota, United States of America; 7 Institute for Global Change Biology, School for Environment and Sustainability, University of Michigan, Ann Arbor, Michigan, United States of America; Sun Yat-Sen University, CHINA

## Abstract

Grasslands are integral to maintaining biodiversity and key ecosystem services and are under threat from climate change. Plant and soil microbial diversity, and their interactions, support the provision of multiple ecosystem functions (multifunctionality). However, it remains virtually unknown whether plant and soil microbial diversity explain a unique portion of total variation or shared contributions to supporting multifunctionality across global grasslands. Here, we combine results from a global survey of 101 grasslands with a novel microcosm study, controlling for both plant and soil microbial diversity to identify their individual and interactive contribution to support multifunctionality under aridity and experimental drought. We found that plant and soil microbial diversity independently predict a unique portion of total variation in above- and belowground functioning, suggesting that both types of biodiversity complement each other. Interactions between plant and soil microbial diversity positively impacted multifunctionality including primary production and nutrient storage. Our findings were also climate context dependent, since soil fungal diversity was positively associated with multifunctionality in less arid regions, while plant diversity was strongly and positively linked to multifunctionality in more arid regions. Our results highlight the need to conserve both above- and belowground diversity to sustain grassland multifunctionality in a drier world and indicate climate change may shift the relative contribution of plant and soil biodiversity to multifunctionality across global grasslands.

## Abstract 2

Grasslands are integral to maintaining biodiversity and key ecosystem services under climate change. This study combines a global survey of 101 grasslands with a novel microcosm experiment to show that plant and soil microbial diversity independently predict a unique portion of variation in above- and below-ground functioning.

## Introduction

Grasslands are one of the major biomes of the world, covering about 40% of the Earth’s surface and 69% of the Earth’s agricultural land area [1–3]. These ecosystems are globally recognized for their native and high biodiversity [4,5] and are also essential for the provisioning of a wide range of ecosystem services, including food production [2], carbon (C) storage and climate mitigation [6], pollination [7], water regulation, and a range of cultural services [8]. Despite their importance, grasslands are increasingly under threat of degradation at an alarming pace in many parts of the world due to climate change [8–10]. For example, approximately 49% grassland ecosystems experience degradation, of which climate change accounted for approximately 45.5% of degradation [11]. Part of this degradation is associated with the loss of their biodiversity. Furthermore, above- and belowground biodiversity are tightly linked, with plants providing energy belowground via plant litter and root exudates, and soil microorganisms supporting nutrient availability for plants through fixation and organic matter (OM) decomposition [12–14]. However, there is little understanding on how plant and soil biodiversity interact under environmental stresses to regulate the provision of multiple ecosystem functions (multifunctionality) in grasslands across contrasting environmental conditions.

The positive relationship between plant diversity and ecosystem functioning has been a focus of research for more than 2 decades [15–19], with similar findings recently expanded to belowground communities in terrestrial ecosystems across biomes [20,21]. In both cases, the higher the plant or soil biodiversity, the higher the ecosystem multifunctionality supported by terrestrial ecosystems. Further, the effects of plant and microbial diversity are largely expected to be complementary [22], maximizing rates of key above- and belowground services [23]. However, our current knowledge and lack of strong experimental evidence does not allow us to tease apart the relative contribution, and interactions between plant and soil microbial diversity in driving multifunctionality, as the influence of plant and soil biodiversity are often evaluated in isolation. Moreover, recent studies suggest that plant and soil biodiversity rarely peak in the same locations across the globe [24,25], highlighting an important need for quantification of the relative contribution of plant and soil biodiversity to support grasslands multifunctionality. Such knowledge is critically important to improve prediction on consequences of biodiversity loss for key ecosystem functions and to develop management policies to mitigate those impacts.

Current uncertainties in the relative contribution of plant and soil microbial diversity in regulating multifunctionality in a climate change context exist because of 3 main reasons. First, the interaction between plant and soil biodiversity is often overlooked in global studies and manipulative experiments [21,26–28]. However, this knowledge is critical to understand whether these 2 groups can work independently and/or synergistically in supporting ecosystem functions. For example, soils with high plant and microbial diversity may support higher rates of nutrient cycling and decomposition compared to soils with either low plant or microbial diversity [24] but strong empirical evidence is lacking. Second, most reports on grasslands multifunctionality are based on local and regional field surveys but lack a global study and experimental support from simultaneous manipulation of plant and soil biodiversity [20,29,30]. Such global field survey observations are required to establish the generality of biodiversity–ecosystem functioning relationships and to understand the relative contribution of above- and belowground diversity in a real-world scenario and how it is influenced by key abiotic (e.g., soil types) and climatic (e.g., aridity) conditions. In contrast, mechanistic evidence from manipulative experiments that simultaneously quantify the influence of plant and soil microbial diversity in driving ecosystem multifunctionality is needed to distinguish between statistical correlation and causal relationships. However, manipulative experiments that simultaneously account for plant and microbial diversity were not feasible until recently, mainly due to a technical inability to maintain soil biodiversity gradients over a sustained period in the presence of plant communities [31]. In addition, biodiversity effects on ecosystem functions can be modified by community composition and abundance, and it is important to distinguish the differential effects of biodiversity (i.e., richness, composition, and abundance). Finally, the current rate of biodiversity loss is associated with significant change in climatic conditions but there is a lack of experimental evidence on how climate change variables impact the relationship between plant and soil microbial diversity and ecosystem multifunctionality in the context of ongoing global environmental changes [32]. Addressing these knowledge gaps is critical to advance our understanding of plant–soil feedback effects, predict the consequences of current environmental disturbances, and to develop effective management and conservation policies to maintain and restore global grasslands.

**Fig 1:**
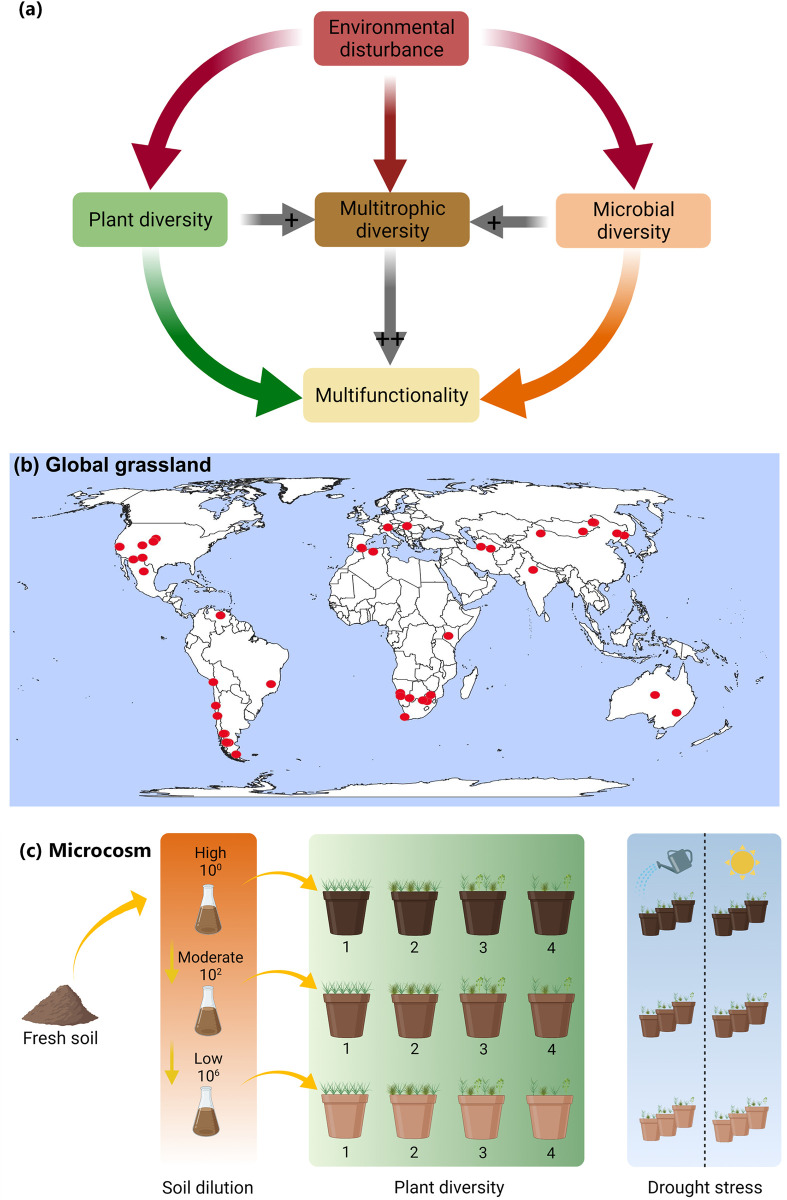
Experimental framework to study the effects of plant and microbial diversity interactions on ecosystem multifunctionality. (a) Conceptual framework exploring the relative contribution of plant and microbial diversity and their combination, i.e., multitrophic diversity to multiple ecosystem functions; (b) locations of the 101 sites included in the global grassland survey; (c) full-factorial design of the microcosm study comprising four plant diversity levels of plant species and 3 microbial diversity levels obtained from a dilution-to-extinction approach. (Fig 1A and 1C were created with BioRender.com, while Fig 1B was created using open source QGIS software version 3.34).

Here, we aimed to (1) quantify the unique and interactive contribution of microbial and plant diversity in driving multiple ecosystem functions in global grasslands; and (2) to assess the influence of observational (aridity) and experimental (drought) climatic conditions on the linkages between biodiversity and functions. We chose aridity and drought treatments because both represent changes in water availability and potentially induce similar community responses, and both are expected to intensify under projected climate change [33]. High plant diversity may promote fertility via contributions of high quantity and diversity of inputs, which drive microbial processes such as decomposition, mining and priming that require a highly diverse microbial community. Similarly, a high microbial diversity per se may not be enough to promote all components of multifunctionality, as diverse resources (e.g., litter or roots exudates) from aboveground producers may promote microbial diversity and activities that promote soil fertility, decomposition, and productivity in terrestrial ecosystems. For these reasons we hypothesized that (1) microbial and plant diversity are equally important drivers of ecosystem multifunctionality due to their differential roles in supporting different functions (i.e., producers versus decomposers). (2) Plant and soil microbial richness explain a unique portion of total variation in the ecosystem multifunctionality across global grasslands under climate change, supporting the argument that plant and soil biodiversity have a complementary effect on ecosystem functions (Fig 1A).

To achieve this, we combined a global field survey including plant and soil biodiversity and multiple functions in 101 grasslands across biomes (from arid to tropical grasslands; Fig 1B) with a novel manipulative experiment of plant and soil biodiversity using 4 levels of plant richness and 3 levels of soil microbial richness in a full-factorial design subjected to drought stress (Fig 1C). The main aim of our manipulative experiment was to determine the direct effects and relative contribution of plant and soil biodiversity in explaining multifunctionality under contrasting water availability conditions. Insights from soil microbial diversity–ecosystem functioning studies are constrained by substantial limitations, particularly survey-based studies that link natural variation in microbial communities to change in ecosystem functions. That is because disentangling cause and effect from observational (survey-based) studies is difficult as other drivers (e.g., temperature, soil structure, chemistry) could simultaneously impact both community and ecosystem functions [34]. To account for these, our experimental framework for the manipulative experiments used one soil type to avoid confounding impacts of soil characteristics such as structure and nutrients availability. By using a dilution-to-extinction approach, all parameters of soils remained the same except for a change in soil biodiversity, allowing the identification of direct impacts of soil biodiversity on ecosystem functions [28,35]. For this study, we chose functions that were directly linked to ecosystem productivity and nutrient cycling and were grouped into decomposition, soil nutrient pools, and plant production [17].

Our unique approach combining 2 independent methods (observational and experimental) provides a complementary assessment of the linkages between plant and soil microbial diversity and ecosystem multifunctionality from local to global scales. However, it should be noted that the objective of this study was not to directly compare, or merge, these 2 data sets (global and microcosm) which have important methodological differences (e.g., measured functions). Rather, we aimed to assess whether the critical role of biodiversity remains consistent in both, despite these data sets having different drivers (e.g., large number of soil types, climatic conditions, and range of vegetation diversity in the global data set). Our results identify, for the first time, the complementary roles of plant and soil biodiversity in explaining multiple aspects of functions in grasslands under experimental climate change and across environmental gradients.

## Results

### Plant and soil microbial richness are linked to ecosystem multifunctionality across global grasslands

**Fig 2:**
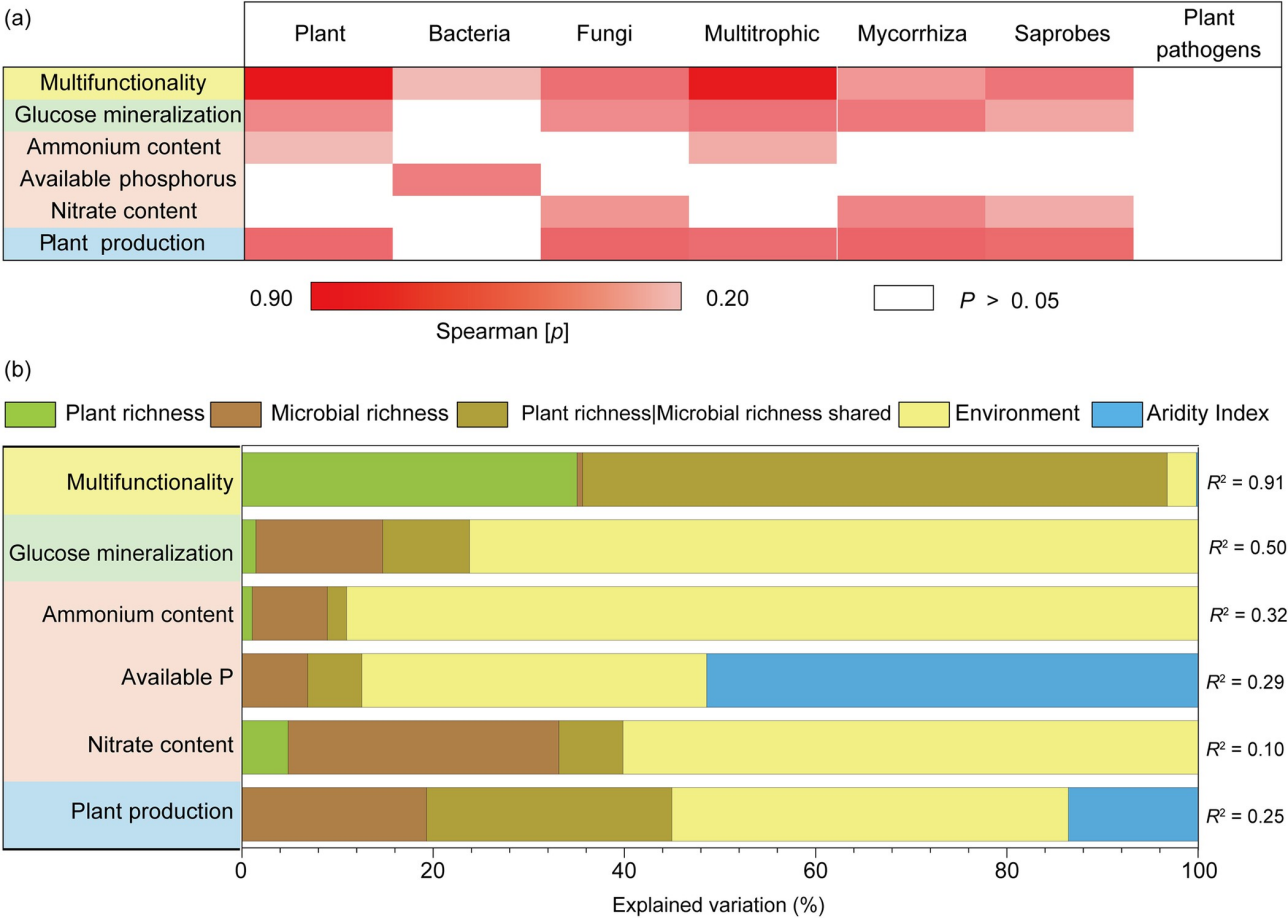
Links between ecosystem multifunctionality and plant and soil microbial richness in the grassland field survey. (a) Significant correlations (Spearman; *p* ≤ 0.05) between the richness of groups of organisms and weighted multifunctionality and individual ecosystem functions. *P*-values showing Spearman correlations in Table E/1 in S1 Text. (b) Variation partitioning modeling was used to evaluate the unique and shared portions of variation in ecosystem properties explained by plant richness, microbial richness, environment, and aridity index. Plant richness | microbial richness shared refers to the percent of shared variation in ecosystem properties explained by plant and microbial richness. *P*-values associated with the unique portions explained by different groups of predictors are available in Table F/1 in S1 Text. The contribution of plant and microbial (bacteria and fungi) richness, environment (distance from the equator, plant cover, soil pH, % clay, soil C, and mean annual temperature) and aridity index to weighted multifunctionality and individual functions (glucose mineralization—soil respiration with glucose addition; soil inorganic pools—ammonium content, available P, nitrate content; plant productivity—net primary production). Microbial richness corresponds to a composite metric of their joint richness (standardized between 0 and 1) and environment properties correspond to a standardized principal component analysis first axis obtained from the multiple properties. *R*^2^ values express total variances corresponding to model adj. Plant pathogen only includes fungal pathogens. The data underlying this figure can be found in S1 Data.

Correlation analyses showed that above- and belowground biodiversity were significantly associated with ecosystem functions across global grasslands (Fig 2A and Fig G panels (a)–(c) in S1 Text). We found significantly positive relationships between multifunctionality with plant, bacterial, fungal, and multitrophic richness (Fig 2A and Table E/1 in S1 Text). Furthermore, there were strong positive correlations between multifunctionality and richness of saprophytic fungi (Fig 2A). We further showed that multifunctionality was significantly correlated with plant richness in both arid and hyperarid environments, with fungal richness (including mycorrhizal and saprotrophic fungi) in humid and arid environments, and with bacterial richness in dry subhumid and semiarid environments (Fig H in S1 Text).

To gain insight into the level of performance of multiple functions in response to biodiversity, we then evaluated the correlation between biodiversity and an increasing number of ecosystem functions crossing different levels (multi-threshold approach) of functions (from low to high levels of function). Our results showed that the diversity of bacteria and multitrophic (all microbes + plants) diversity (i.e., richness) were positively correlated with an increasing number of functions at a relatively low level of function (i.e., 10% and 25% threshold relative to maximum observed level of function). Richness of fungi (as well as mycorrhizal and saprotrophic fungi) was positively correlated with an increasing number of functions at relatively low and medium levels of functions (i.e., 10%, 25%, and 50% thresholds relative to maximum observed level of function). Plant richness was positively correlated with an increasing number of functions at a relatively low level of function (i.e., 10% threshold) (Fig I/1 in S1 Text), supporting the strongest association with the 26% threshold. Multitrophic richness indexes were associated with an increasing number of functions supporting relatively higher levels of functions compared with individual richness (Fig J/1 in S1 Text).

We used variation partitioning modeling to evaluate the unique and shared portions of variation in ecosystem properties explained by plant richness, microbial richness, environment, and aridity index. Across global grasslands, we found that plant and soil microbial richness significantly explained a unique (35% and 1%, respectively) and shared (61%) portion of variation in multifunctionality, with environmental variables alone explaining 3% of this variation (Fig 2B and Table F/1 in S1 Text). Remarkably, soil microbial richness explained important unique fractions of the variation in individual ecosystem functions, such as glucose mineralization (13%), soil inorganic pools (43%), and plant production (19%), while plant diversity had no significant impact on these (Fig 2B). Our results also showed that environmental properties (50.8%) explained, on average, more variation in ecosystem functions than plant richness (7.0%), microbial richness (12.7%), and combination of both (18.5%). The aridity index only predicted significant variation in available phosphorus (51%), with no significant prediction for others.

We then used structural equation modeling (SEM) to evaluate the direct link between plant and soil biodiversity and weighted multifunctionality in the global data set. Our results provided solid evidence that plant and fungal richness played a significant role in multifunctionality after accounting for environmental properties of spatial influence, climate, and soil variables (Fig 4A and Fig L in S1 Text). Plant and fungal richness were directly and positively correlated with multifunctionality, whereas microbial and plant richness mediated environmental properties effect on multifunctionality. Specifically, distance equator and mean ambient temperature (MAT) indirectly impacted multifunctionality via their effect on plant richness. The impacts of aridity index on multifunctionality were direct and indirect via soil pH and fungal richness (the higher the aridity index the lower the aridity; Fig 4A). We also found nonlinear relationships between aridity index and multifunctionality, a positive correlation when the aridity index was less than 0.32 (Fig K in S1 Text).

### Assessing the relative contribution of plant and soil microbial richness as multifunctionality drivers in a microcosm study

Linear mixed modeling showed that plant and soil microbial richness had independent effects on multifunctionality in the experimental microcosm study (Tables G/1-G/2 and Fig F in S1 Text). The decrease in microbial richness had negative effects on both above- and belowground functions, such as leaf nitrogen (N) content, plant productivity (green canopy cover and plant height), soil nutrient storage (soil C, N, and phosphorus (P)), and soil inorganic pools (inorganic N), while it increased (positive effect) only leaf P content and soil dissolved organic C (DOC). Increasing plant richness decreased leaf C content, whereas it had positive effects on belowground functions such as total dissolved N (TDN), soil nutrient storage (soil P), and OM decomposition (glucose mineralization). The drought event significantly increased soil nutrient storage (total N and total P) and consequently, multifunctionality.

**Fig 3:**
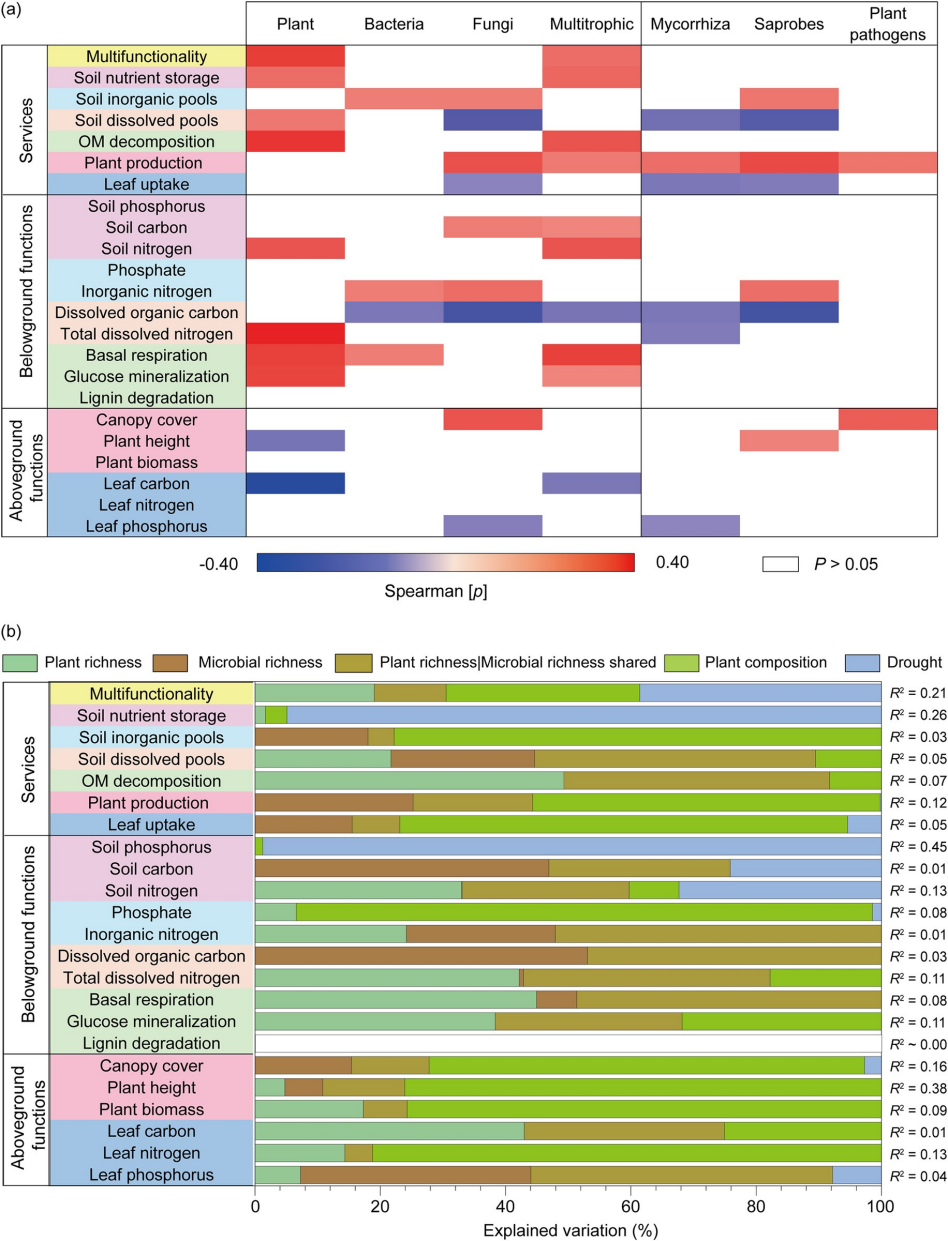
The contribution and relationship of plant and soil biodiversity to weighted multifunctionality in the microcosm experiment. (a) Significant correlations (Spearman; *p* ≤ 0.05) between richness groups and weighted multifunctionality, services and individual belowground and aboveground functions. The different background colors are used to highlight which individual functions belong to services categories. Plant and microbes richness corresponds to a composite metric of their joint diversity (standardized between 0 and 1). Fungal phylotypes include mycorrhiza, saprotrophs, and plant pathogens fungi. *P*-values showing Spearman correlations in Table E/2 in S1 Text. (b) Variation partitioning modeling was used to evaluate the unique and shared portions of variation in ecosystem properties explained by plant richness, microbial richness, plant composition, and drought. Plant richness | microbial richness shared refers to the percent of shared variation in ecosystem properties explained by plant and microbial richness. *P*-values associated with the unique portions explained by different groups of predictors are available in Table F/2 in S1 Text. Plant pathogen only includes fungal pathogens. The data underlying this figure can be found in S1 Data.

Spearman correlations showed a significant positive relationship between multifunctionality (obtained from 6 grouped functions) and plant and multitrophic (plant x microbe) richness (Fig 3A and Fig G panels (d)–(f) and Table E/1 in S1 Text) but not microbial diversity alone. Multitrophic richness was responsible for maintaining a positive relationship with belowground functions, specifically soil nutrient storage and OM decomposition (Fig 3A). In particular, we found a positive relationship between plant richness and soil N stocks (TDN and total N), and OM decomposition (basal respiration and glucose mineralization), while fungal richness was positively and negatively related to C stocks (total C) and labile C (DOC), respectively (Fig 3A). We then tested the biodiversity-multi-threshold multifunctionality relationship and found a positive association between plant and multitrophic richness and functions at high thresholds of over 50% and 90% of their maximum observed levels of functioning, respectively (Fig I/2 in S1 Text).

**Fig 4:**
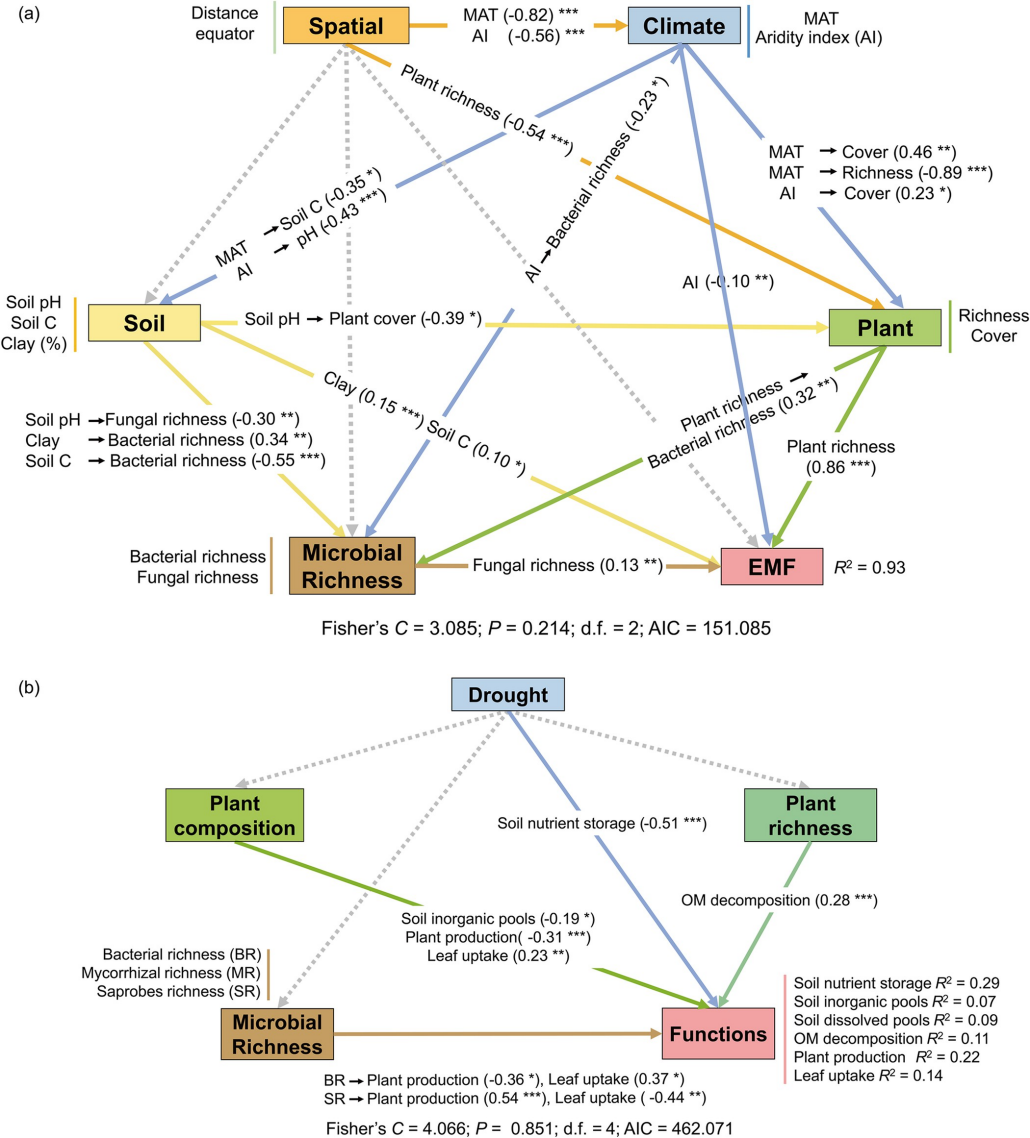
Direct and indirect drivers of ecosystem functions. Piecewise structural equation models for (a) weighted multifunctionality derived from the global grassland survey (*n* = 101) and for (b) above- and belowground functions derived from the microcosm study (*n* = 157). We aimed to identify the direct relationship between plant and soil biodiversity (bacterial and fungal richness) and multiple ecosystem functions. Soil microbial richness was included as a composite variable, including information of soil bacterial and fungal taxa. Please note, the higher the aridity index the less arid environment it represents. Numbers adjacent to arrows are indicative of the effect size of the relationship. Dashed lines indicate that none of the variables included was significant (*p*-value >0.05). For each model, the proportion of variance explained (*R*^2^) and the various goodness-of-fit statistics are shown below the response variables. Significance levels are as follows: *p ≤ 0.05, **p ≤ 0.01, and ***p ≤ 0.001. AIC, Akaike information criterion; EMF, ecosystem multifunctionality; MAT, mean annual temperature. Information about a priori models can be found Tables E/1 and E/2 in S1 Text. The data underlying this figure can be found in S1 Data.

To distinguish richness effects on multifunctionality from community variables, we used partial correlations and found that the positive effects of plant richness on multifunctionality were marginally affected by plant composition and abundance (r = 0.14; *p* = 0.086). Despite positive impact of microbial richness on some individual functions, there was a lack of significant effects of bacterial and fungal richness on multifunctionality which remained unchanged when controlling for microbial abundance and community composition (Table H in S1 Text). Thus, we included plant composition (i.e., different plant combinations), together with plant and microbial richness and drought to assess their importance and contribution as predictors of multifunctionality, and individual functions. Variation partitioning modeling showed that plant and soil microbial diversity independently predict a unique portion of variation in above- and belowground functioning, suggesting that both types of biodiversity complement each other (Fig 3B and Table F/2 in S1 Text). Specifically, plant richness primarily affected belowground functions, such as soil nutrient storage (i.e., soil N), soil dissolved pools (i.e., total dissolved N), and OM decomposition, while microbial richness explained aboveground plant production and leaf uptake (i.e., leaf P) (Fig 3B). Aboveground functions, on average, were predicted by microbial richness (20%), plant combination (63%), and drought (2.5%). Belowground functions, on average, predicted by plant richness (18.3%), microbial richness (10.3%), plant combination (25%), and drought (23.8%) (Fig 3B). Furthermore, plant richness (19%), along with its shared portion with microbial richness (11%), explained the important variation in multifunctionality after accounting for plant composition (31%) and drought disturbance (39%) (Fig 3B). Drought significantly predicted soil nutrient storage, particularly for soil P (Fig 3B). SEM further showed that drought negatively affected ecosystem function, e.g., soil nutrient storage (Fig 4B and Fig M in S1 Text). We found that above- and belowground biodiversity has complementary effects on ecosystem functions. More specifically, plant richness positively affects OM decomposition, bacterial richness drives leaf uptake, and saprobes richness positively affects plant production.

## Discussion

By combining 2 independent approaches including a global grassland survey and a unique manipulative microcosm experiment, our study showed that: (1) plant and soil microbial diversity independently predict a unique portion of total variation in above- and belowground ecosystem functioning, suggesting that both plant and soil biodiversity complement each other to support functions across global grasslands subjected to climate change (aridity gradients and drought); and (2) multitrophic biodiversity, i.e., plant and soil microbial richness combined, is positively associated with multifunctionality (e.g., plant production, nutrient cycling, soil fertility), and was essential to maintaining key ecosystem services such as soil nutrient storage and primary production globally. This highlights the multiple pathways in which plants and soils can interact to support soil fertility and nutrient cycling, and also plant productivity.

We further illustrated that at a global level, grassland multifunctionality varied from a stronger positive association with fungal richness in humid regions, to a stronger positive association with plant richness in hyperarid regions, while in arid regions both plant and fungal richness were strongly associated with multifunctionality. In addition, aridity indirectly explained global multifunctionality by a negative association with soil C and soil texture (i.e., percentage clay), while the drought event implemented experimentally only temporarily impacted multifunctionality in the microcosm study, which substantially recovered. Instead, the experimental drought event led to an increase in soil nutrient storage, particularly soil P concentration in the soil [36], likely caused by temporary reduced microbial demand and nutrient uptake by plants in response to drought event [37,38]. Together with climate change linked intensification of aridity and drought, these findings support the necessity in maintaining key ecosystem functions through maintenance of biodiversity in vulnerable ecosystems which are projected to face increasing water scarcity under climate change conditions.

Our global survey showed that the richness of soil fungal groups, particularly mycorrhizal and saprotrophic fungi, were strongly and positively associated with ecosystem multifunctionality across grasslands. We chose to split total fungal community into saprophytic, mycorrhizal, and pathogen groups because these groups play very different role in ecosystem functions. For example, mycorrhizal fungi are known to promote a range of benefits to plants by providing mineral nutrients and protection from abiotic and biotic stresses [39–41], while pathogen have detrimental impact on plant health and productivity [42]. Saprotrophic fungi are essentially soil decomposers capable of breaking down organic matter otherwise unavailable to plant growth [43], due to their extensive hyphal networks [44] and their capacity to break down more recalcitrant forms of organic matter [45]. Thus, fungal communities provide critical support for both above- and belowground communities and functions. Additionally, the positive relationship between multitrophic richness and multifunctionality was weaker in comparison to individual richness groups. In fact, positive effects of plant richness on multifunctionality at a global level were a result of its indirect effects on microbial richness, as previously observed across biomes (e.g., forests, grasslands, and shrublands) [21,28], suggesting plant and microbial richness have selective effects when driving specific ecosystem services. For example, our microcosm study demonstrated a strong, positive, and significant association of multitrophic richness with soil C and N storage and OM decomposition, whereas the importance of plant production was highlighted in the global survey. This is supported by the microcosm study where the promotion of soil N stocks by plant richness could be attributed to N inputs being made available by increasing microbial activity and more labile forms of OM decomposition. In contrast, fungal richness promoted C stocks and consumed labile C (DOC), suggesting the higher C storage observed in soils with high microbial richness was likely driven by fungal retention, possibly from photosynthates derived from higher green canopy cover, combined with low temperature and types of soil texture limiting C decomposition [46].

Further, our results suggested that the strongest positive association between fungal richness and multifunctionality was observed at low and medium thresholds (i.e., <50% threshold relative to maximum observed level of function) across global grasslands. While in the microcosm study, an association between plant richness and functions operating at medium and high levels of functioning (>50% threshold) was observed. These results indicate that fungal and plant diversity are both necessary to sustain a wide range of rate/availabilities of multiple functions, supporting a fundamental level of functioning in grasslands. The use of the multiple threshold approach is important here in identifying different aspects of ecosystem functioning and their drivers. For example, low thresholds reflect an ecosystem’s ability to provide many functions at moderate levels, emphasizing broad functionality and resilience. In this context, fungal richness contributes significantly to sustaining multifunctionality, as fungi play crucial roles in nutrient cycling and soil structure maintenance [21]. On the other hand, high thresholds highlight an ecosystem’s capacity to excel in a few functions, which often involves specialized adaptations and resource use efficiencies [47]. In particular plants, as primary producers, directly affect resource availability and biomass production [48], and thus are expected to have a significant impact in the provisioning and stability of multiple services [20,29]. The fact that plant richness and plant composition in the microcosm study explained independently a unique portion of variation of multifunctionality demonstrated that both richness and composition, of plant species are important drivers of ecosystem functions [49,50].

Our study provides new insights on the fundamental importance of plant and soil biodiversity to support grassland multifunctionality in a context of climate change, and water availability, in particular. Both our data sets illustrate that the impacts of reduced water availability, i.e., aridity in global natural grasslands and a single experimental drought event in the microcosm study, influence certain aspects of ecosystem multifunctionality. However, our findings also suggest that globally, the decline in soil fertility under increasing aridity and increasing temperatures will markedly influence multifunctionality. Thus, indirect effects of aridity are likely to have major effects on multifunctionality. For example, the decline of C that we observed with aridity could be associated with the decline in plant productivity and plant cover and the likely decrease in plant-derived organic inputs into the soil [51].

The sharp decline observed in the fungal richness, namely saprotrophic and mycorrhizal fungi, from hyperarid regions could be a response to high mean annual temperatures and the decline of vegetation. In fact, Berdugo and colleagues [51] refers to progressive decline phases in ecosystem functioning as a response to aridity until an irreversible phase is reached. In response to increasing aridity, vegetation is expected to be impacted first, followed by soil fertility, and only then microbial communities such as saprotrophic fungi, known to be key drivers of soil fertility [52], and mycorrhizal fungi linked to plant composition and nutrient cycling [53]. Our experimental study illustrated an almost complete recovery of multifunctionality, likely because it only included a single extreme drought event. More frequent drought disturbances would probably have more permanent and deleterious effects in both above- and belowground functions, constraining biodiversity–multifunctionality relationships to recover [54]. We expectedly found some difference in results from our 2 independent data sets such as the relative contributions of microbial diversity on multifunctionality between global survey and experimental studies given restrictions of space for root foraging and microbial dispersal in pots. These differences can be explained as these data sets have different drivers and design (e.g., gradient of soil types, climatic conditions, diversity of plant species). However, key findings from 2 data sets, regarding critical roles of biodiversity, overlap supporting our hypotheses. We should also like to note that all ecological methodologies have some limitations and ours is not an exception. For example, dilution-to-extinction approach, despite considered to be the best microbial diversity manipulation approach, can cause some shift in community composition.

Overall, we provide novel empirical evidence that maintaining both plant and microbial diversity is crucial to sustain multiple ecosystem functions due to their independent and interactive roles in ecosystem functioning under climate change. In particular, we showed that plant and soil biodiversity explained a unique proportion of total variation in the distribution of multifunctionality across global grasslands and in experiments subjected to climatic stress. These findings support that plant and soil biodiversity complement each other to catalyze functioning under environmental stress as both high plant and soil biodiversity can lead to supporting more functions at a higher level. Fungal richness had a direct impact on multifunctionality in global grasslands while plant richness effects were indirectly driven by microbial richness, particularly for aboveground functions. Multitrophic richness was vital in maintaining belowground functions, particularly soil nutrient storage and OM decomposition but also plant productivity globally. We provide novel insights that fungal richness has a stronger positive association with multifunctionality in humid regions while plant richness in hyperarid regions and both in arid regions, indicating context dependency in biodiversity and ecosystem functions relationships [55]. Overall, our study provides empirical evidence and supports that both above- and belowground diversity and their interactions in terrestrial ecosystems should be explicitly considered in future ecosystem biodiversity and functioning studies and in developing new management, restoration, and conservation policies for grasslands.

## Material and methods

### Global survey

#### Study sites

We used composite topsoil samples from global field surveys, which were conducted between 2014 and 2019 following standardized field protocols [25,56,57]. We included 101 grasslands from 6 continents, providing a large representation of all climatic grassland biomes on the planet, i.e., humid, dry subhumid, semiarid, arid, and hyperarid (Fig 1B). Grasslands were dominated by globally dominant grass genera such as *Festuca*, *Stipa*, *Andropogon*, *Bouteloua*, *Cynodon*, *Eragrostis*, *Poa*, *Sporobolus*, and *Trachypogon*. These grasslands also included a wide range of environmental conditions such as soil pH (4.3 to 8.6), fine texture (1.7% to 83.6%), carbon (0.4 to 215.1 g C kg soil^-1^), mean annual precipitation (26 to 1,471 mm), and temperature (−2.7 to 27.2°C). Perennial plant richness and plant cover were determined in the field using the line-intercept method according to Maestre and colleagues [17]. Aridity index is calculated as Mean Annual Precipitation divided by Potential Evapotranspiration as defined by UNEP 1992. Similarly, UNEP 1992 defined different aridity index categories as follows: (1) dry subhumid (0.5 ≤ AI < 0.65); (2) semiarid (0.2 ≤ AI < 0.5); (3) arid (0.05 ≤ AI < 0.2), and (4) hyperarid (AI < 0.05).

Composite soil (top, approximately 10 cm) samples (from 5 to 10 soil cores) were collected in these locations following the protocol described in Maestre and colleagues [17] aiming to capture the within-location heterogeneity variation in soil environmental conditions. A portion of these soils was frozen (−20°C) after sampling for molecular analyses, while another portion was air-dried and used for determining soil properties.

#### Microbial diversity characterization

Bioinformatic analysis on the 16S rDNA/18S rDNA amplicons was performed to characterize bacterial and fungal diversity across global grasslands [21,57]. Briefly, pair-end reads were merged using USEARCH [58], followed by a quality filtering step on the merged reads with expected error (ee) set as 1.0 maximum. High-quality reads were then dereplicated and singletons were removed. The zOTUs (zero-radius operational taxonomic units; denoised sequences, 100% sequence identity) were gained by denoising (error-correction) the amplicon reads using unoise3 [59]. Representative sequences of 16S rDNA and ITS zOTUs were annotated against the Silva [60] database in QIIME [61] using the UCLUST algorithm [58], respectively. Prior to diversity calculation, a normalization procedure was performed at 10,000 and 4,000 reads per sample for 16S and 18S, respectively. Alpha diversity indices, including richness and Shannon diversity, and beta diversity indices including Bray–Curtis matrices were calculated in QIIME. The FungalTrait database was employed to gain the functional guilds information of fungi [62].

#### Ecosystem functions

We selected 5 ecosystem functions from those available in the global survey to match those functions available from the microcosm study. These 5 functions were further grouped into 3 ecosystem services: OM decomposition (glucose induced respiration), soil inorganic pools (availability of nitrate, ammonium, and phosphate), and plant production (net primary productivity; NPP). The 5 functions considered are mostly weakly correlated with each other (Table D/2 in S1 Text), supporting their inclusion in the multifunctionality index. We used NDVI (normalized difference vegetation index), from MODIS satellite imagery (https://modis.gsfc.nasa.gov) (2001 to 2020; 250 m resolution), as our proxy of NPP. The content of nitrate and ammonium were determined as a proxy of nitrogen availability as described in Maestre and colleagues [17]. The availability of these 2 N forms is associated with important processes such as nitrification and ammonification rates. The content of soil phosphate was determined using Olsen P approach as described in Maestre and colleagues [17]. Soil respiration in response to glucose was determined at 20°C and 60% water holding capacity (WHC) using Microresp as described in Campbell and colleagues [63].

### Microcosm study

#### Experimental design

The microcosm study was conducted at Western Sydney University (WSU) for approximately 6 months in a full-factorial design, including 4 levels of plant richness, 3 levels of microbial diversity, a drought event at the end (well-watered control versus drought), and 6 replicates for each combination of treatments. Soils were collected from the experimental grassland research facility at WSU. Briefly, each plant species (originally 6; see plant diversity manipulation) was represented in monoculture, in 3 different 3 species combinations, and 1 final combination of 6 species (1, 3, 6 species). In addition, 18 control replicates with no plants and with only the microbial diversity treatment were established, totaling 198 microcosms. Plant richness was monitored throughout the experimental period: a preliminary assessment was made in the first 12 weeks of plant establishment (T1); after 6 weeks of plant establishment and before drought was initiated (T2); at the end of the 2-week drought (T3) and after 5-weeks from the end of drought, i.e., drought recovery (T4). After drought recovery, all above- and belowground functions were recorded. Since there was some plant mortality throughout the study, the replication per treatments varied and the observed plant richness was considered at the end (1, 2, 3, 4 species; Table A in S1 Text) for posterior analyses, in a total of 157 microcosms.

The experiment was carried out in a glasshouse room with corresponding Spring and Summer temperatures from monthly averages (Table B in S1 Text), from September to January, based on the last 10-year monthly average data (i.e., 28/18°C day/night) from Meteorological Bureau Station 067021 (http://www.bom.gov.au). During the months of plant establishment and as natural light subsidized, 650W LED LumiGrow Pro lights were used to promote plant growth. Microcosms were watered with sterile water every 24 to 48 h to maintain soil moisture in the range of 11% to 14% (corresponding to 60% to 80% WHC of the soil) throughout the experiment.

#### Soil microbial diversity manipulation

Soil was initially collected from the top 0 to 15 cm at the Yarramundi paddock research site, WSU in Richmond, NSW, Australia (33°36’34.2"S, 150°44’20.9"E). The soil is characterized as loamy sand, with a particle distribution of 81.3% sand, 7.5% silt and 11.2% clay content, and a volumetric WHC of 15% to 20%. It is also characterized by low organic C (1%), total N (0.1%), and pH 5.7. Full soil characteristics can be found in Churchill and colleagues [64].

After field collection, soil was sieved with a 5-mm sieve and aliquots of approx. 1.8 kg of fresh soil were stored in plastic bags. The bags containing soil were sterilized using gamma radiation (50 kGy each) at ANSTO Life Sciences facilities, Sydney, Australia. Gamma radiation was used as it is known to cause minimal change to the physical and chemical properties of soils compared with other methods such as autoclaving [65,66]. A separate portion of soil was kept aside to serve as inoculum and original microbial community characterization. The dilution-to-extinction approach was then used to prepare soil inoculum [28,67]. Six different replicates of inoculum from the unsterilized soil were produced to create a serial dilution to avoid pseudo-replication. The parent inoculum suspensions were prepared by manually mixing 1:4 ratio of soil in sterilized 1× phosphate buffer saline (PBS) for 5 min. The sediment was then allowed to settle for approx. 2 min, and 1:100 serial dilutions were prepared from the suspension using a plate with magnetic stirring to mix the contents homogenously. From these serial dilutions, only 3 diversity levels were selected as microbial inoculum, depicting high (HD = 10^0^), moderate (MD = 10^−2^), and low diversity (LD = 10^−6^). The microbial inoculation consisted of a 1:9 ratio of inoculum to soil, leading to soil moisture of approx. 18% to 20% (corresponding to previously determined WHC). Bags were kept closed with a cotton plug to allow gas exchange and incubated in the dark at room temperature (20°C) for 18 weeks to allow microbial colonization and biomass to recover. The bags were gently mixed every 3 weeks to homogenize microbial growth. Microcosm inoculation and posterior setup after 18 weeks of incubation was carried within a laminar flow hood and all parts used for the inoculation were sterilized by autoclaving at 121°C. Microcosms consisted of pots (inner diameter = 14 cm, height = 15 cm; volume = 1.9 L) filled with an average of 1.6 kg of soil (dry mass) with the selected microbial diversity levels. After 18 weeks of inoculation, before experimental setup, and compared to undiluted soil (HD = 10^0^), microbial richness of MD was reduced by 9% for bacteria and 14% for fungi and microbial richness of LD was reduced by 52% for bacteria and 73% for fungi.

#### Plant diversity manipulation

Six plant species were initially selected based on species classification into 3 different functional groups, according to their intrinsic physiological and morphological differences. The species used comprised C4 grasses (*Chloris gayana*, *Digitaria eriatha*), C3 grasses (*Lolium perenne*, *Phalaris aquatica*), and legumes (*Biserrula pelecinus*, *Medicago sativa*). Cultivar information can be found in Churchill and colleagues [64]. All species were represented in the monocultures (*N* = 6) and 3 species combination (*N* = 3) were initially set up as X (*Chloris gayana*, *Lolium perenne*, and *Biserrula pelecinus*), Y (*Lolium perenne*, *Phalaris aquatica*, and *Medicago sativa*), Z (*Chloris gayana*, *Biserrula pelecinus*, and *Medicago sativa*), with all 6 species represented in a final combination (*N* = 1).

Seedlings of at least 3 cm in height were previously germinated in sterilized vermiculite in growth cabinets (25/15°C day/night; 14/12 h day/night) before transplanting them into pots with soil microbial dilutions in the greenhouse. All pots aimed to have 6 plants, but establishment rate varied. Briefly, monocultures aimed to have 6 seedlings, 3 species combinations aimed to have 2 seedlings per species, and 6 species combination pots aimed to have 1 seedling per species, to maintain similar evenness. Due to initial low establishment rate, transplant attempts were maintained for the first 3 months and thus it was not possible to account for similar germination times between all species and replicates. Plant diversity (Shannon–Wiener index; H’) was recorded at T2 and maintained throughout the duration of the experiment (Fig A in S1 Text). Seedlings less than 3 cm height as well as dead plants after drought were excluded from plant richness counts and function measurements. In the end, the legume species *Biserrula pelecinus* had the highest mortality and lowest transplant survival and for this reason was removed from further analyses. After plant establishment, the final plant richness achieved was 1, 2, 3, and 4 species from combinations of *Chloris gayana*, *Digitaria eriatha*, *Lolium perenne*, *Phalaris aquatica*, and *Medicago sativa*.

#### Drought manipulation

A two-week drought treatment was applied at the 16th week of plant establishment to all plant–microbial diversity interactions and no plant controls. Here, half of the initial 6 replicates being maintained under a reduced watering regime and the other half at a well-watered regime (*n* = 3). For drought replicates, watering was maintained to achieve a soil moisture in the range of 5% to 7% by weight (30% to 40% WHC of the soil), whereas well-watered replicates were watered to maintain a moisture content in the range of 11% to 13% (60% to 70% WHC). At the end of the drought event (and after sampling), the drought replicates were brought back to well-watered ranges and allowed to recover for 5 weeks before a final harvest took place.

#### Microbial diversity characterization

Soil was collected for characterization of soil diversity by amplicon sequencing using an Illumina MiSeq platform by Next-Generation Sequencing Facility at the WSU (Richmond, NSW, Australia). Soil genomic DNA was extracted using the PowerSoil DNA Isolation kit (Qiagen, United States of America) according to the manufacturer’s instructions, with modification of the soil weight used (0.50 g) and the initial cell-lysis step, using a FastPrep-24 5G bead beating system (MP Biomedicals, California, USA) at a speed of 5.5 m s^-1^ for 30 s. Bacterial 16S rRNA and fungal ITS2 region at the start and end of the study were sequenced using 341F/805R and fITS7/ITS4 primer sets, respectively [21]. To quantify the total abundance of bacteria and fungi in the soil at the start and end of the study, the 16S rRNA gene (primer set Eub518-Eub238) and ITS region (primer set ITSIf-5.8s), respectively, were quantified in a Light Cycler 96 Real Time PCR System (Roche) as described in [68].

Bioinformatic analysis on the 16S rDNA/ITS amplicon reads was performed as previously described for global samples. Approximately 15.8M and 17.1 high-quality merged sequences were mapped for 16S rDNA and ITS zOTUs, respectively. Prior to diversity calculation, a normalization procedure was performed at 15,000 and 11,000 sequences per sample for 16S rDNA and ITS table, respectively. Alpha diversity indices, including richness, and beta diversity indices, including Bray–Curtis matrices, were calculated in QIIME.

At the end of the experiment, on average, bacterial communities were dominated by Actinobacteria, followed by Proteobacteria, and Firmicutes; fungal communities were dominated by Ascomycota. We used in this study, microbial richness (number of soil phylotypes) as a metric of soil biodiversity since richness is the simplest and most used metric of biodiversity. The fungal functional groups such as soil mycorrhizal, saprotrophic and plant fungal pathogens were identified using FungalTraits [69]. The dilution-to-extinction approach had a significant effect on reducing the soil microbial richness and composition (Figs B (panel a), C, and D in S1 Text and Table C in S1 Text). There was no significant effect of plant richness on total soil bacterial and fungal richness treatment at the end of the experiment; however, plant richness had a significant effect on fungal composition (Fig C in S1 Text). In the microbial richness levels, mycorrhizal fungi decreased on average 76% from HD to MD and LD, saprotrophic fungi decreased 9% and 61% to MD and LD respectively, and plant pathogens decreased 7% and 30% to MD to LD, respectively.

#### Ecosystem functions

We quantified 16 above- and belowground ecosystem functions regulated by both plant and soil microorganisms and representing direct measures of biotic/abiotic processes as well as process indicators such as nutrient pools [70]. These functions contribute to climate regulation and support primary production, soil fertility, nutrient cycling, and photosynthesis. We have grouped them into 6 service categories: soil nutrient storage (total C, N, P), soil inorganic pools (inorganic N, phosphate), OM decomposition (basal respiration, glucose, mineralization, lignin degradation), soil dissolved pools (dissolved organic C (DOC), total dissolved N (TDN)), plant production (plant biomass (shoot and root) biomass, height, green canopy cover), and leaf uptake (leaf C, N, P content) (Fig F in S1 Text). We used substrate induced-respiration measurements as indicators of OM decomposition. Total dissolved N represents the pool of organic and inorganic N. The inclusion of soil dissolved C and N pools provide a more dynamic interpretation of C and N cycles since they represent stocks directly linked to process rates. Similarly, total C, N, P stocks as indicators of nutrient storage are also the result of biological processes and have been found to regulate plant production and diversity in desertification reversal [30,71] (Qiu and colleagues). The 16 variables considered are mostly weakly correlated with each other (Table D/1 in S1 Text). Posteriorly, individual functions were grouped into service categories also having weak correlation with each other, further supporting their inclusion in the multifunctionality index and services (Table D/2 in S1 Text). All functions and services are also positively correlated to the multifunctionality index (except for a negative correlation between multifunctionality and canopy cover and leaf N, which are nonsignificant), facilitating the interpretation of the results. At the final harvest, plant height was determined for each individual plant from the ground up and community weighted means were used for 2-, 3-, and 4-plant species combinations to obtain an average value per pot. Then, shoots were cut at the soil surface, the number of individuals of each species was counted, and shoot per species and total root biomass per pot were determined, by obtaining dry mass after oven-dry at 60°C for 72 h. Green canopy cover per pot was obtained using Canopeo [72], which provides a measure of “greenness” of the vegetation, and thus acts as a proxy of plant productivity. Leaf and soil C and N were determined on a Vario El Cube CHNS Elemental Analyser (Elementar Australia Pty) and leaf and soil P were determined using analytical Epsilon 3 EDXRF spectrometer (Malvern Panalytical, Leyweg, Almelo, the Netherlands) from ground oven-dried samples (soil: 40°C and leaves: 60°C; for 72 h) at the end of the experiment. Leaf C, N, and P were obtained per species within each plant combination and community weighted means were used. Dissolved organic C and TDN were determined on a total organic C analyser fitted with a total N measurement unit (TOC-L TNM-L, Shimadzu, Sydney, Australia) after a filtered extraction with 0.05M K_2_SO_4_. The availability of inorganic N (sum of ammonium and nitrate) and phosphate (PO_4_^3-^) concentrations in the soil were determined on a SEAL AQ2 Discrete Analyser (SEAL Analytical, USA) after extraction with 2M KCl and 0.5M HCl, respectively. Basal respiration, lignin degradation, and glucose mineralization were determined following the MicroResp approach to measure lignin and glucose-induced respiration [63]. Substrate-induced respiration of glucose and lignin are calculated as respiration in glucose or lignin less the basal respiration. All raw data are made available at Figshare- (DOI: 10.6084/m9.figshare.20077217) and included as a S1 Data file of the paper, and sequence data are available at European Nucleotide Archive with accession number PRJEB53575.

### Statistical analysis

#### Multifunctionality index

To obtain a quantitative multifunctionality index, 3 independent multifunctionality approaches were used: (1) the weighted multifunctionality index; (2) the multi-threshold multifunctionality index; and (3) multiple individual functions. The weighted ecosystem multifunctionality index was used to prevent the up-weighting of certain aspects of ecosystem functioning or services since some functions were not accounted for between the 2 data sets, thus providing insight into the average biodiversity effect on services [47]. It was obtained by first standardizing all individual ecosystem functions between 0 and 1 (rawFunction − min(rawFunction)/(max(rawFunction) − min(rawFunction)); functions were transformed (logarithm or square root) when necessary before standardization, grouped as ecosystem services by averaging individual functions belonging to each group in each data set (3 services were considered in the global survey and 6 services in the microcosm study), and then all services were averaged to account for equal service contribution. We tested the linear relationship between weighted ecosystem multifunctionality and averaged multifunctionality (Fig G panel (b) in S1 Text). The *R*^2^ was greater than 0.9 with a significant fit, thus, weighted multifunctionality was used in the main text and referred as multifunctionality.

For biodiversity indexes (microbial richness: combination of bacteria and fungi; plant x microbe richness: combination of plant and soil microbes, i.e., multitrophic richness), a similar standardization and weighting was applied using the richness of individual groups so that the richness of each group contributed equally to each biodiversity metric. The multi-threshold approach was included since it provides insight into the level of performance of multiple functions in response to biodiversity [73], by estimating the relationship between biodiversity and the number of functions (rate or availability) that simultaneously exceed a critical threshold (>10%, 25%, 50%, 75%, and 90% of the maximum observed level of functioning for a given function).

#### Linear mixed modeling

We used a linear mixed effect model approach fitted by restricted maximum likelihood in the microcosm study to test whether soil microbial diversity interacts with plant richness to influence the multiple functions considered. All plant and soil microbial characteristics and all 16 ecosystem functions were assessed for variation among plant and microbial richness and drought treatments after a recovery period. Observed plant richness was considered as a continuous variable (co-variate) due to variation throughout the study. Plant combination (presence/absence of species) was used as a random effect to account for variation from the different plant species established within each combination. Due to low replication at 4-species combination at final harvest, we additionally tested the response of all functions to 3-species diversity, supporting the robustness of our findings. The general response of ecosystem functions and services did not change. When necessary, data were transformed (logarithm or square root) to improve assumptions of normality of errors and homogeneity of variance. Treatment effects were considered statistically significant at *p* < 0.05 and a Tukey HSD post hoc test was used for multiple pairwise comparisons. Linear mixed modeling was performed with JMP v16.0.0 (SAS Institute). A nonmetric multidimensional ordination (NMDS) was applied on the matrix of bacterial and fungal composition at the zOTU level using the Bray–Curtis distance and obtained with the package Vegan from R [74] and a two-way PERMANOVA was applied for treatment effect test.

#### Multi-model inference based on information theory

For the microcosm study, a model-averaging procedure [75] was employed for multifunctionality, ecosystem services, and individual functions separately. This analysis was based on minimizing the corrected Akaike information criterion (AICc) to evaluate the % of importance of the predictors under consideration, namely plant richness and composition and microbial richness and drought as drivers of multifunctionality. We included plant composition in the microcosm study analysis to account for the lack of similar germination times between all species and replicates which can contribute to variation in final plant composition rates. All predictors and response variables were standardized before analyses to interpret parameter estimates on a comparable scale. This method is similar to a variance partition analysis because of previous standardization of predictors [76–79]. We removed microbial composition as well as plant and soil microbial richness combined as predictors due to high correlation (Table D/3 in S1 Text). Microbial richness was obtained from the mean of standardized bacterial and fungal diversity, so each group contribution is accounted equally. Plant species composition was estimated with presence (1) and absence (0), we then conducted unconstrained PCoA with Jaccard distance analysis. Model residuals were inspected to ensure for constant variance and normality. The importance of predictors was expressed as the percentage of variance they explain, based on the comparison between the absolute values of their standardized regression coefficients and the sum of all standardized regression coefficients from all predictors in the models. R^2^ values presented express total variances corresponding to model adj. R^2^ obtained from parameter estimates averaged across all models.

A similar approach was applied for the global survey where aridity index was included and environmental properties (distance from the equator, plant cover, soil pH, % clay, soil C, and mean annual temperature) corresponded to a standardized first axis of a PCA analysis obtained from the multiple properties (Fig E (panel b) in S1 Text). We did not include plant composition for the global grassland survey because obtaining absolute abundance of plant species at the global scale was not possible. We excluded the multitrophic richness as predictors in both data sets due to high correlation (Table D/3 in S1 Text). Multi-model analyses were carried out using SAM 4.0 [80].

#### Spearman and partial correlations

In both data sets, the relationship between the standardized individual richness groups (plant richness, bacterial richness, fungal richness, mycorrhizal, saprotrophic, and plant pathogens fungal richness) and multitrophic richness (joint effects of plant and soil microbial richness) with ecosystem multifunctionality (weighted and multi-threshold multifunctionality) was tested using Spearman correlations. For the microcosm study, to test for the influence of community composition and abundance in biodiversity–multifunctionality relationships, we conducted partial correlation analysis between plant and soil biodiversity and weighted multifunctionality, accounting for plant abundance (biomass) and microbial abundance (quantitative PCR data) and plant composition (main axis of a PCA analysis) and microbial composition (main axis of an NMDS analysis). Regression analysis, Spearman correlations, and partial correlations were performed with JMP v16.0.0 (SAS Institute).

### Variation partitioning modeling (VPA)

VPA was used to quantify the relative importance of plant richness, microbial richness, environment, and aridity index in global survey. Environmental properties (distance from the equator, plant cover, soil pH, % clay, soil C, and mean annual temperature) corresponded to a standardized first axis of a PCA analysis obtained from the multiple properties (Fig E (panel b) in S1 Text). VPA also was used to evaluate the unique and shared portions of variation in ecosystem properties explained by plant richness, microbial richness, plant composition, and drought in microcosm experiment. We used the *varpart* function from the vegan package in R package to perform these analyses. Note that adjusted coefficients of determination in multiple regression/canonical analysis can, on occasion, take negative values. Negative values in the variance explained for a group of predictors on a given response variable are interpreted as zeros [21].

### Structural equation modeling (SEM)

We used piecewise SEM to evaluate the direct link between plant and soil biodiversity and weighted multifunctionality in the global survey, after accounting for multiple key environmental factors such as spatial influence (distance from equator), climate (mean annual temperature and aridity), plant (richness and cover), and soil (soil pH, total C content, and percentage of clay) attributes. We also used piecewise SEM to evaluate the effects of plant, bacterial, and fungal richness on above- and belowground functions after accounting for influences of drought and plant composition in the microcosm study. Drought was included as a categorical variable with 2 levels: 1 (well-watered) and 0 (drought). All remaining variables were included as independent observable variables, with plant composition consisting of the main axis of a PCA analysis obtained from the final % of composition of each plant species. We considered that plant richness influenced final plant composition by the end of the experiment since plant composition was dependent and thus derived from the experimentally manipulated plant richness. Due to the duration of microcosm study, we also considered that by the end, there would be an influence of microbial richness on the plant composition since this metric was derived from final shoot biomass. These analyses were conducted using “piecewiseSEM,” “lme4,” “nlme,” and “QuantPsyc” packages. We used the Fisher’s *C*-test (when 0.05 < *P* < 1.00) to confirm the goodness of the modeling results.

## Supporting information

S1 Text Supporting Information. **Fig A in S1 Text.** Plant diversity (Shannon–Wiener Index; *H*’) throughout the duration of the microcosm study (approx. 6 months) for each sampling point (T1, T2, T3, T4). T1-T2 corresponds to plant diversity establishment, T2-T3 corresponds to drought disturbance (2 weeks), and T3-T4 corresponds to a recovery phase (5 weeks). Values correspond to mean ± s.e. HD, high soil diversity; MD, moderate soil diversity; LD, low soil diversity. Different lower case letters indicate significant differences (*p* < 0.05) between time points. The data underlying this figure can be found in S1 Data. **Fig B in** S1 **Text.** The effects of the dilution-to-extinction approach in the microcosm study, on (a) soil bacterial and fungal (*p* < 0.0001) richness (no. phylotypes) and (b) on soil bacterial and fungal relative gene abundance at the start and at the end of the experiment (approx. 6 months). Values correspond to mean ± s.e. HD, high soil diversity; MD, moderate soil diversity; LD, low soil diversity. Different lower case letters indicate significant differences (*p* < 0.05) for each separate time point. Gene relative abundance at the start was obtained from a subsample (*n* = 18) in comparison to end of experiment (*n* = 157). The data underlying this figure can be found in S1 Data. **Fig C in** S1 **Text.** The effects of the dilution-to-extinction approach in the microcosm study, on (a) soil bacterial and fungal richness (no. phylotypes) and mycorrhizal, saprotrophic and plant pathogens fungi richness (*p* < 0.001) as well as (b) bacterial and fungal composition summarized from a nonmetric multidimensional ordination (NMDS) with a stress level <0.2 based on no. of phylotypes. Values in (a) correspond to mean ± s.e. HD, high soil diversity; MD, moderate soil diversity; LD, low soil diversity. Numbers 1, 2, 3, 4 correspond to different plant richness at the end of the experiment. Different lower case letters indicate significant differences (*p* < 0.05). The data underlying this figure can be found in S1 Data. **Fig D in** S1 **Text.** The taxonomic composition of bacteria and fungi (per phylum; %) in microcosm study following a dilution-to-extinction approach, at the start and end of the experiment (*n* = 175; approx. 6 months). HD, high soil diversity; MD, moderate soil diversity; LD, low soil diversity. Numbers 0, 1, 2, 3, 4 correspond to plant richness. The data underlying this figure can be found in S1 Data. **Fig E in** S1 **Text.** Principal coordinate analysis (PCoA) of (a) plant species composition (shoot biomass) at final harvest in the microcosm study, and principal components analysis (PCA) of (b) environmental properties (distance from the equator, plant cover, soil pH, % clay, soil C, and mean annual temperature (MAT)) in the global survey. The data underlying this figure can be found in S1 Data. **Fig F in** S1 **Text.** The effects of plant richness and microbial richness obtained from a dilution-to-extinction approach on aboveground and belowground ecosystem functions, in the microcosm study. Values correspond to mean ± s.e. Statistical significance can be found in Table F/2 in S1 Text. HD, high soil diversity; MD, moderate soil diversity; LD, low soil diversity. Ecosystem function units: dissolved organic C, total dissolved N, inorganic N, phosphate (mg/kg); soil and leaf C, N, P, and green canopy cover (%); basal respiration, glucose mineralization, and lignin degradation (μgCO_2_-C/g/h); plant height (cm); plant biomass (g). The data underlying this figure can be found in S1 Data. **Fig G in** S1 **Text.** Relationships between (a, d) weighted and (b, e) averaged multifunctionality and different standardized biodiversity groups considered in the global survey and microcosm study (richness of plant, bacteria, fungi, microbes, plant x microbes and fungal phylotypes of mycorrhizal, saprotrophic and plant pathogens). For the weighted multifunctionality index, ecosystem functions were previously averaged into ecosystem services before multifunctionality is calculated, so that functions from each ecosystem service are equally accounted for its contribution to multifunctionality, whereas in the case of averaged multifunctionality index, individual functions were equally averaged. (c, f) Linear relationship between weighted ecosystem multifunctionality and averaged multifunctionality. Adjusted R^2^ values are shown, and significance level is indicated by * *p* < 0.05, ** *p* < 0.01, and *** *p* < 0.001. The data underlying this figure can be found in **S1 Data. Fig H in S1 Text.** Relationships between weighted multifunctionality and plant and microbial richness in the global grassland survey for each aridity index. Microbial groups encompass total bacteria and fungi as well as fungal phylotypes of mycorrhizal, saprotrophic fungi, and plant pathogens. Plant x microbial richness corresponds to a composite metric of their joint diversity (standardized between 0 and 1). Weighted multifunctionality relationship with richness groups is obtained by best fitted regressions (linear or quadratic) for each aridity index (humid, dry subhumid, semiarid, arid, hyperarid). Significance level is indicated by * *p* < 0.05, ** *p* < 0.01, and *** *p* < 0.001. The table below presents all the adjusted R^2^ values corresponding to the best fit and corresponding *p*-value. The data underlying this figure can be found in S1 Data. **Fig I/1 in** S1 **Text.** Relationships between multiple threshold multifunctionality and plant and microbial richness in the global grassland survey. Microbial groups encompass total bacteria and fungi as well as fungal phylotypes of mycorrhizal, saprotrophic fungi, and plant pathogens. Plant x microbial richness corresponds to a composite metric of their joint diversity (standardized between 0 and 1). Multiple thresholds functioning relationship with richness groups is obtained by best fitted regressions (R^2^ values presented correspond to the best fit—linear or quadratic) between the richness of different groups of organisms and the number of functions above multiple thresholds. Adjusted R^2^ values are shown when significant. Significance level is indicated by * *p* < 0.05, ** *p* < 0.01, and *** *p* < 0.001. The data underlying this figure can be found in S1 Data. **Fig I/2 in** S1 **Text.** Relationships between multiple threshold multifunctionality and plant and microbial richness in the microcosm study. Microbial groups encompass total bacteria and fungi as well as fungal phylotypes of mycorrhizal, saprotrophic fungi, and plant pathogens. Plant x microbial richness corresponds to a composite metric of their joint diversity (standardized between 0 and 1). Multiple thresholds functioning relationship with richness groups is obtained by best fitted regressions (R^2^ values presented correspond to the best fit—linear or quadratic) between the richness of different groups of organisms and the number of functions above multiple thresholds. Adjusted R^2^ values are shown when significant. Significance level is indicated by * *p* < 0.05, ** *p* < 0.01, and *** *p* < 0.001. The data underlying this figure can be found in S1 Data. **Fig J/1 in** S1 **Text.** Diversity effects for a range of ecosystem multiple services thresholds in the global grassland survey. The dotted curves indicate the changes in the number of services per unit increment of diversity of plant (A), microbial (C), and multitrophic (E). Effects of plant (B), microbial (D), and multitrophic (F) diversity on the number of services above thresholds. Lines represent the slope between soil microbial diversity and the number of services greater than or equal to a threshold value ranging from 5% to 99% of the maximum for each service. The data underlying this figure can be found in S1 Data. **Fig J/2 in** S1 **Text.** Diversity effects for a range of ecosystem multiple services thresholds in the microcosm study. The dotted curves indicate the changes in the number of services per unit increment of diversity of plant (A), microbial (C), and multitrophic (E). Effects of plant (B), microbial (D), and multitrophic (F) diversity on the number of services above thresholds. Lines represent the slope between soil microbial diversity and the number of services greater than or equal to a threshold value ranging from 5% to 99% of the maximum for each service. The data underlying this figure can be found in S1 Data. **Fig K in** S1 **Text.** Nonlinear relationships between multifunctionality and aridity index. The data underlying this figure can be found in S1 Data. **Fig L in** S1 **Text.** A priori structural equation modeling (SEM) metamodel aimed to evaluate the link between microbial richness and multifunctionality (EMF) after controlling for key ecological predictors such as spatial, climate, and soil and plant attributes. Different categories of predictors were grouped in the model for graphical simplicity. Aridity = - 1 x Aridity Index. MAT = mean annual temperature. The fitted model is available in Fig 4A. **Fig M in** S1 **Text.** A priori structural equation modeling (SEM) metamodel aimed to evaluate the link between microbial richness and multifunctionality (EMF) after controlling for key ecological predictors such as drought, and plant richness and composition. Different categories of predictors were grouped in the model for graphical simplicity. The fitted model is available in Fig 4B. **Table A in S1 Text.** Experimental design describing replicates per diversity and drought treatments for the microcosm study. **Table B in S1 Text.** Greenhouse temperature (day/night °C) conditions obtained from monthly averages, of September to January, based on 10-year monthly average data from Meteorological Bureau Station 067021 (http://www.bom.gov.au) for the microcosm study. **Table C in S1 Text.** Statistical summary (degrees of freedom, F ratio, *p*-values) for the microcosm study, and R2 adj of linear mixed model of microbial richness and microbial gene abundance (bacteria: 16S rRNA; fungi: ITS region) at final harvest, when accounting for plant combination variability (*n* = 157); 4-plant richness (1, 2, 3, 4) and 3-plant richness (1, 2, 3) was considered as a co-variate, separately. *P-*values in bold represent significant differences between fixed-effects (*p* < 0.05). **Table D/1 in S1 Text.** Spearman correlation coefficient (ρ) among the 16 functions, and with the weighted multifunctionality index, for the microcosm study. Abbreviations: DOC, dissolved organic C; TDN, total dissolved N; S_C, Soil C; S_N, Soil N; S_P, Soil P; InN, Inorganic N; Phos, Phosphate; Water, Basal respiration; Glucose, Glucose mineralization; Lignin, Lignin degradation; C_cover, Canopy cover; P_height, Plant height; P_biom, Plant biomass; L_C, Leaf C; L_N, Leaf N; L_P, Leaf P; EMF, weighted multifunctionality. Significance is shown in bold (* *p* < 0.05, ** *p* < 0.01, and *** *p* < 0.001). **Table D/2 in S1 Text.** Spearman correlation coefficient (ρ) among ecosystem service categories and weighted multifunctionality for global survey and microcosm study. Significance is shown in bold (* *p* < 0.05, ** *p* < 0.01, and *** *p* < 0.001). Coefficients among individual functions and inorganic pools service and weighted multifunctionality for the global survey were included. **Table D/3 in S1 Text.** Spearman correlation coefficient (ρ) among diversity and composition groups and drought for the microcosm study. Plant composition was obtained from first axis of a PCA and microbial composition from first axis of NMDS. For the global survey, coefficients among diversity and abiotic parameters were also included. Significance is shown in bold (** *p* < 0.01, *** *p* < 0.001). **Table E/1 in S1 Text.** *P*-values arising from Spearman correlations in Fig 2A. Significance is shown in bold (*p* < 0.05). **Table E/2 in S1 Text.** *P-*values arising from Spearman correlations in Fig 3A. Significance is shown in bold (*p* < 0.05). **Table F/1 in S1 Text.** *P*-values arising from variation partitioning modeling in Fig 2B. Significance is shown in bold (*p* < 0.05). **Table F/2 in S1 Text.** *P*-values arising from variation partitioning modeling in Fig 3B. Significance is shown in bold (*p* < 0.05). **Table G/1 in S1 Text.** Statistical summary (degrees of freedom, F ratio, *p*-values) for the microcosm study, and R^2^ adj of linear mixed model of weighted multifunctionality and ecosystem services when accounting for plant combination variability (*n* = 157); 4-plant richness (1, 2, 3, 4) and 3-plant richness (1, 2, 3) was considered as a co-variate. *P*-values in bold represent significant differences between fixed-effects (*p* < 0.05). **Table G/2 in S1 Text.** Statistical summary (degrees of freedom, F ratio, *p*-values) for the microcosm study, and R^2^ adj of linear mixed model of single ecosystem functions when accounting for plant combination variability (*n* = 157); 4-plant richness (1, 2, 3, 4) and 3-plant richness (1, 2, 3) was considered as a co-variate. *P*-values in bold represent significant differences between fixed-effects (*p* < 0.05). **Table H in S1 Text.** Partial correlations for the microcosm study, between biodiversity and weighted multifunctionality, controlling for microbial abundance (gene abundance) and community composition (first axis of a NMDS) in the case of microbial richness and plant abundance (total biomass) and plant composition (first axis of a PCA) in the case of plant richness. *P*-values in bold represent significant differences (*p* < 0.05) and underline values are considered marginally significant (*p* < 0.1).
(DOCX)

S1 Data Supplementary data. (XLSX)

S1 Code R code for analysis. (TXT)

## Abbreviations

AIC: Akaike information criterion
ee: expected error
MAT: mean ambient temperature
NPP: net primary productivity
NDVI: normalized difference vegetation index
OM: organic matter; PBS, phosphate buffer saline
SEM: structural equation modeling
WHC: water holding capacity
WSU: Western Sydney University
zOTU: zero-radius operational taxonomic unit
