# Supplementary material for: Aboveground and belowground biodiversity have complementary effects on ecosystem functions across global grasslands
Source: PLoS Biol. 2024 Aug 14;22(8):e3002736. doi: 10.1371/journal.pbio.3002736 (PMC11324184; doi:10.1371/journal.pbio.3002736)
Supplement: S1 Text — Fig A in S1 Text. Plant diversity (Shannon–Wiener Index; H’) throughout the duration of the microcosm study (approx. 6 months) for each sampling point (T1, T2, T3, T4). T1-T2 corresponds to plant diversity establishment, T2-T3 corresponds to drought disturbance (2 weeks), and T3-T4 corresponds to a recovery phase (5 weeks). Values correspond to mean ± s.e. HD, high soil diversity; MD, moderate soil diversity; LD, low soil diversity. Different lower case letters indicate significant differences (p < 0.05) between time points. The data underlying this figure can be found in S1 Data. Fig B in S1 Text. The effects of the dilution-to-extinction approach in the microcosm study, on (a) soil bacterial and fungal (p < 0.0001) richness (no. phylotypes) and (b) on soil bacterial and fungal relative gene abundance at the start and at the end of the experiment (approx. 6 months). Values correspond to mean ± s.e. HD, high soil diversity; MD, moderate soil diversity; LD, low soil diversity. Different lower case letters indicate significant differences (p < 0.05) for each separate time point. Gene relative abundance at the start was obtained from a subsample (n = 18) in comparison to end of experiment (n = 157). The data underlying this figure can be found in S1 Data. Fig C in S1 Text. The effects of the dilution-to-extinction approach in the microcosm study, on (a) soil bacterial and fungal richness (no. phylotypes) and mycorrhizal, saprotrophic and plant pathogens fungi richness (p < 0.001) as well as (b) bacterial and fungal composition summarized from a nonmetric multidimensional ordination (NMDS) with a stress level <0.2 based on no. of phylotypes. Values in (a) correspond to mean ± s.e. HD, high soil diversity; MD, moderate soil diversity; LD, low soil diversity. Numbers 1, 2, 3, 4 correspond to different plant richness at the end of the experiment. Different lower case letters indicate significant differences (p < 0.05). The data underlying this figure can be found in S1 Dat [file pbio.3002736.s001.docx]

# **Figure A.** Plant diversity (Shannon-Wiener Index; H’) throughout the duration of the microcosm study (approx. 6 months) for each sampling point (T1, T2, T3, T4). T1-T2 corresponds to plant diversity establishment; T2-T3 corresponds to drought disturbance (2 weeks) and T3-T4 corresponds to a recovery phase (5 weeks). Values correspond to mean ± s.e. HD, high soil diversity; MD, moderate soil diversity; LD, low soil diversity. Different lower-case letters indicate significant differences (*p* < 0.05) between time points. The data underlying this Figure can be found in S1 Data.

# **Figure B.** The effects of the dilution-to-extinction approach in the microcosm study, on (a) soil bacterial and fungal (*p*<0.0001) richness (no. phylotypes) and (b) on soil bacterial and fungal relative gene abundance at the start and at the end of the experiment (approx. 6 months). Values correspond to mean ± s.e. HD, high soil diversity; MD, moderate soil diversity; LD, low soil diversity. Different lower-case letters indicate significant differences (*p* < 0.05) for each separate time point. Gene relative abundance at the start was obtained from a subsample (n=18) in comparison to end of experiment (n=157). The data underlying this Figure can be found in S1 Data.

# **Figure C.** The effects of the dilution-to-extinction approach in the microcosm study, on (a) soil bacterial and fungal richness (no. phylotypes) and mycorrhizal, saprotrophic and plant pathogens fungi richness (*p*<0.001) as well as (b) bacterial and fungal composition summarized from a non-metric multidimensional ordination (NMDS) with a stress level <0.2 based on no. of phylotypes. Values in (a) correspond to mean ± s.e. HD, high soil diversity; MD, moderate soil diversity; LD, low soil diversity. Numbers 1, 2, 3, 4 correspond to different plant richness at the end of the experiment. Different lower-case letters indicate significant differences (*p* < 0.05). The data underlying this Figure can be found in S1 Data.

# **Figure D.** The taxonomic composition of bacteria and fungi (per phylum; %) in microcosm study following a dilution-to-extinction approach, at the start and end of the experiment (n=175; approx. 6 months). HD, high soil diversity; MD, moderate soil diversity; LD, low soil diversity. Numbers 0, 1, 2, 3, 4 correspond to plant richness. The data underlying this Figure can be found in S1 Data.

**
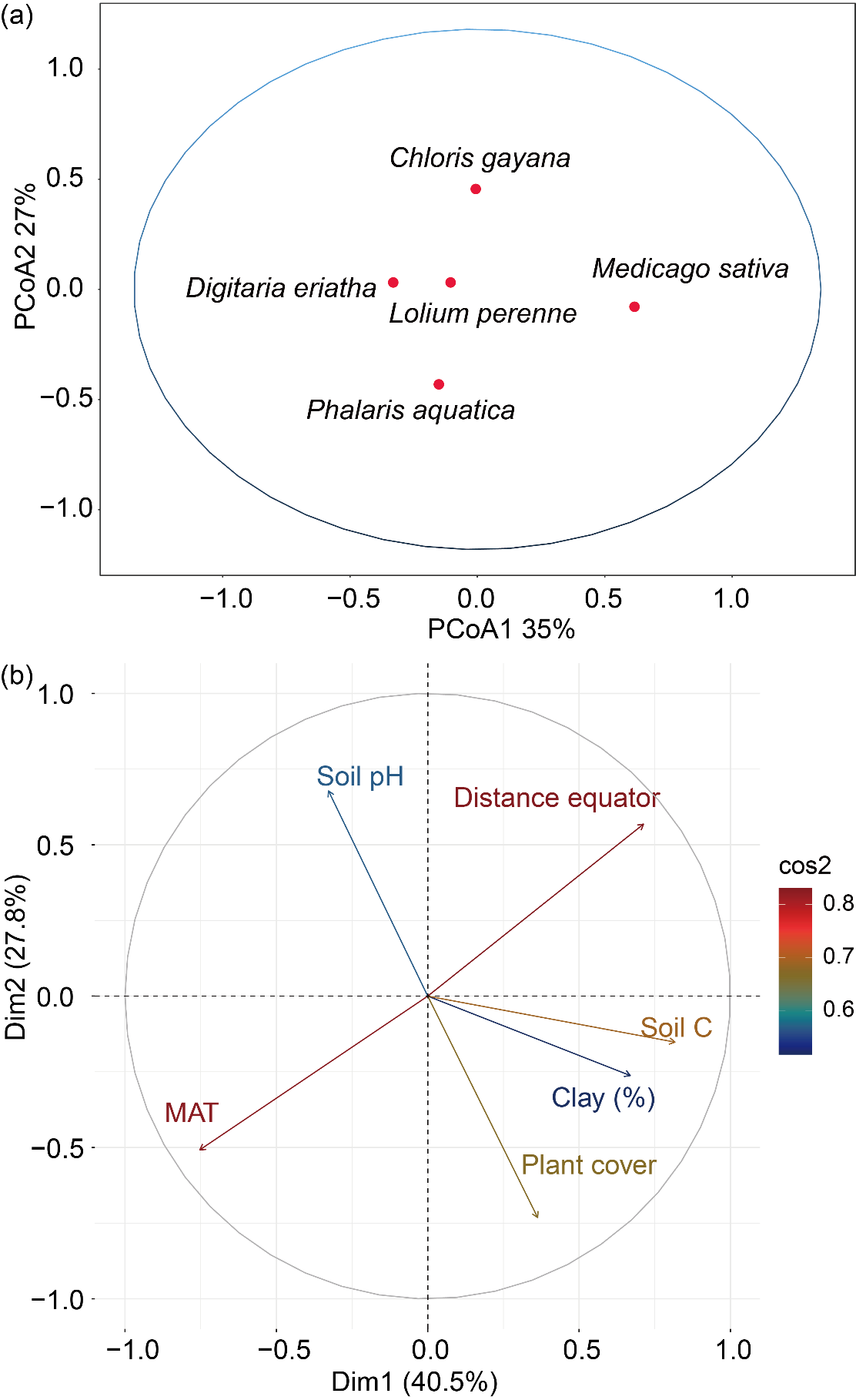
**

# **Figure E.** Principal Coordinate Analysis (PCoA) of (a) plant species composition (shoot biomass) at final harvest in the microcosm study, and principal components analysis (PCA) of (b) environmental properties (distance from the equator, plant cover, soil pH, % clay, soil C and mean annual temperature (MAT)) in the global survey. The data underlying this Figure can be found in S1 Data.

# **Figure F.** The effects of plant richness and microbial richness obtained from a dilution-to-extinction approach on aboveground and belowground ecosystem functions, in the microcosm study. Values correspond to mean ± s.e. Statistical significance can be found in Table S6b. HD, high soil diversity; MD, moderate soil diversity; LD, low soil diversity. Ecosystem function units: dissolved organic C, total dissolved N, inorganic N, phosphate (mg/kg); soil and leaf C, N, P and green canopy cover (%); basal respiration, glucose mineralization and lignin degradation (µgCO_2_-C/g/h); plant height (cm); plant biomass (g). The data underlying this Figure can be found in S1 Data.

# **Figure G.** Relationships between (a, d) weighted and (b, e) averaged multifunctionality and different standardized biodiversity groups considered in the global survey and microcosm study (richness of plant, bacteria, fungi, microbes, plant x microbes and fungal phylotypes of mycorrhizal, saprotrophic and plant pathogens). For the weighted multifunctionality index, ecosystem functions were previously averaged into ecosystem services before multifunctionality is calculated, so that functions from each ecosystem service are equally accounted for its contribution to multifunctionality whereas in the case of averaged multifunctionality index, individual functions were equally averaged. (c, f) Linear relationship between weighted ecosystem multifunctionality and averaged multifunctionality. Adjusted R^2^ values are shown, and significance level is indicated by * *p* < 0.05, ** *p* < 0.01 and *** *p* < 0.001. The data underlying this Figure can be found in S1 Data.

# **Figure H. Relationships between weighted multifunctionality and plant and microbial richness in the global grassland survey for each aridity index.** Microbial groups encompass total bacteria and fungi as well as fungal phylotypes of mycorrhizal, saprotrophic fungi and plant pathogens. Plant x microbial richness corresponds to a composite metric of their joint diversity (standardized between 0 and 1). Weighted multifunctionality relationship with richness groups is obtained by best fitted regressions (linear or quadratic) for each aridity index (humid, dry sub-humid, semiarid, arid, hyperarid). Significance level is indicated by * *p* < 0.05, ** *p* < 0.01 and *** *p* < 0.001. The table below presents all the adjusted R^2^ values corresponding to the best fit and corresponding *p*-value. The data underlying this Figure can be found in S1 Data.

# **Figure I/1.** **Relationships between multiple threshold multifunctionality and plant and microbial richness in the global grassland survey.** Microbial groups encompass total bacteria and fungi as well as fungal phylotypes of mycorrhizal, saprotrophic fungi and plant pathogens. Plant x microbial richness corresponds to a composite metric of their joint diversity (standardized between 0 and 1). Multiple thresholds functioning relationship with richness groups is obtained by best fitted regressions (*R*^2^ values presented correspond to the best fit – linear or quadratic) between the richness of different groups of organisms and the number of functions above multiple thresholds. Adjusted *R*^2^ values are shown when significant. Significance level is indicated by * *p* < 0.05, ** *p* < 0.01 and *** *p* < 0.001. The data underlying this Figure can be found in S1 Data.

# **Figure I/2. Relationships between multiple threshold multifunctionality and plant and microbial richness in the microcosm study.** Microbial groups encompass total bacteria and fungi as well as fungal phylotypes of mycorrhizal, saprotrophic fungi and plant pathogens. Plant x microbial richness corresponds to a composite metric of their joint diversity (standardized between 0 and 1). Multiple thresholds functioning relationship with richness groups is obtained by best fitted regressions (R^2^ values presented correspond to the best fit – linear or quadratic) between the richness of different groups of organisms and the number of functions above multiple thresholds. Adjusted R^2^ values are shown when significant. Significance level is indicated by * *p* < 0.05, ** *p* < 0.01 and *** *p* < 0.001. The data underlying this Figure can be found in S1 Data.

**
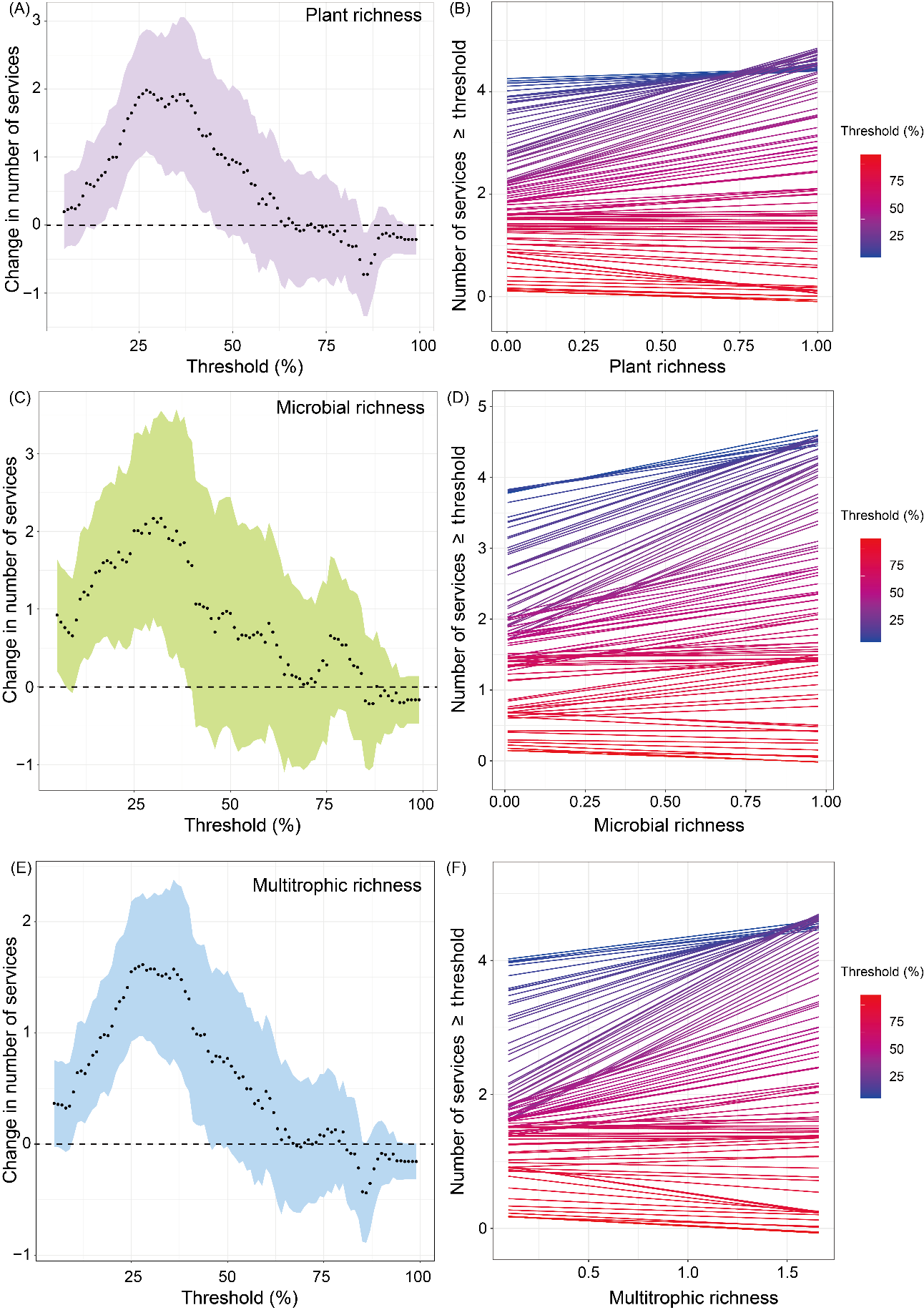
**

# **Figure J/1. Diversity effects for a range of ecosystem multiple services thresholds in the global grassland survey.** The dotted curves indicate the changes in the number of services per unit increment of diversity of plant (A), microbial (C), and multitrophic (E). Effects of plant (B), microbial (D), and multitrophic (F) diversity on the number of services above thresholds. Lines represent the slope between soil microbial diversity and the number of services greater than or equal to a threshold value ranging from 5 to 99% of the maximum for each service. The data underlying this Figure can be found in S1 Data.

**
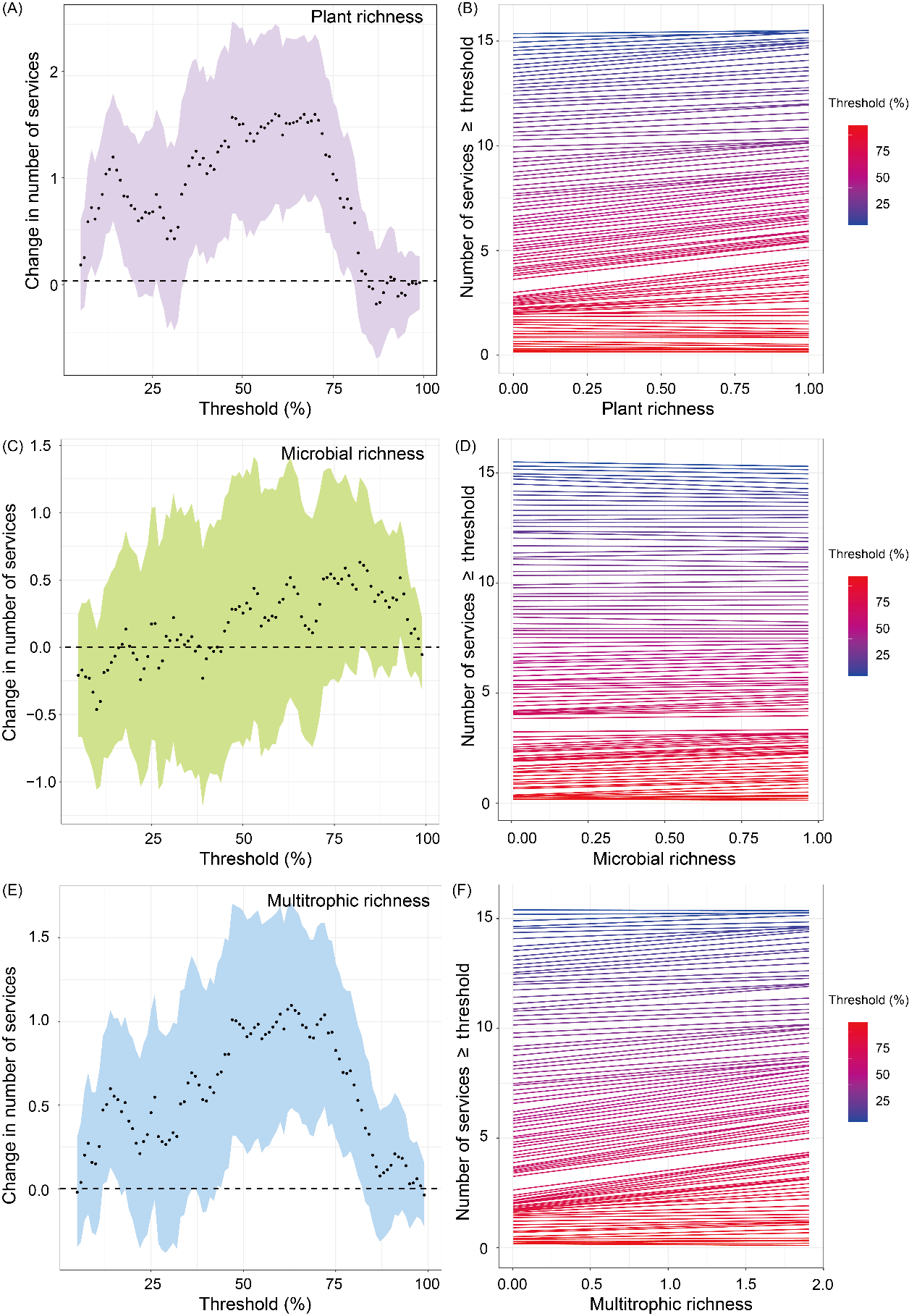
**

# **Figure J/2. Diversity effects for a range of ecosystem multiple services thresholds in the microcosm study.** The dotted curves indicate the changes in the number of services per unit increment of diversity of plant (A), microbial (C), and multitrophic (E). Effects of plant (B), microbial (D), and multitrophic (F) diversity on the number of services above thresholds. Lines represent the slope between soil microbial diversity and the number of services greater than or equal to a threshold value ranging from 5 to 99% of the maximum for each service. The data underlying this Figure can be found in S1 Data.

**
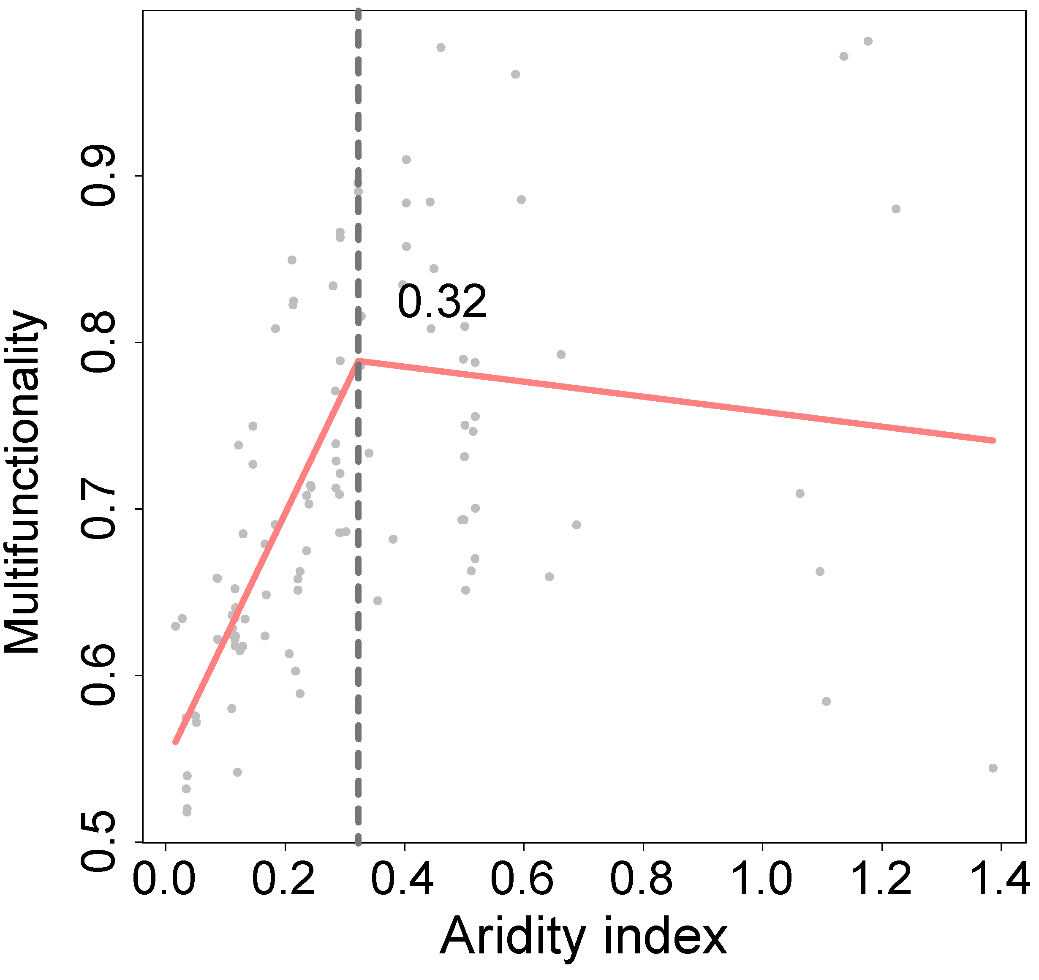
**

# **Figure K. Nonlinear relationships between multifunctionality and aridity index.** The data underlying this Figure can be found in S1 Data.


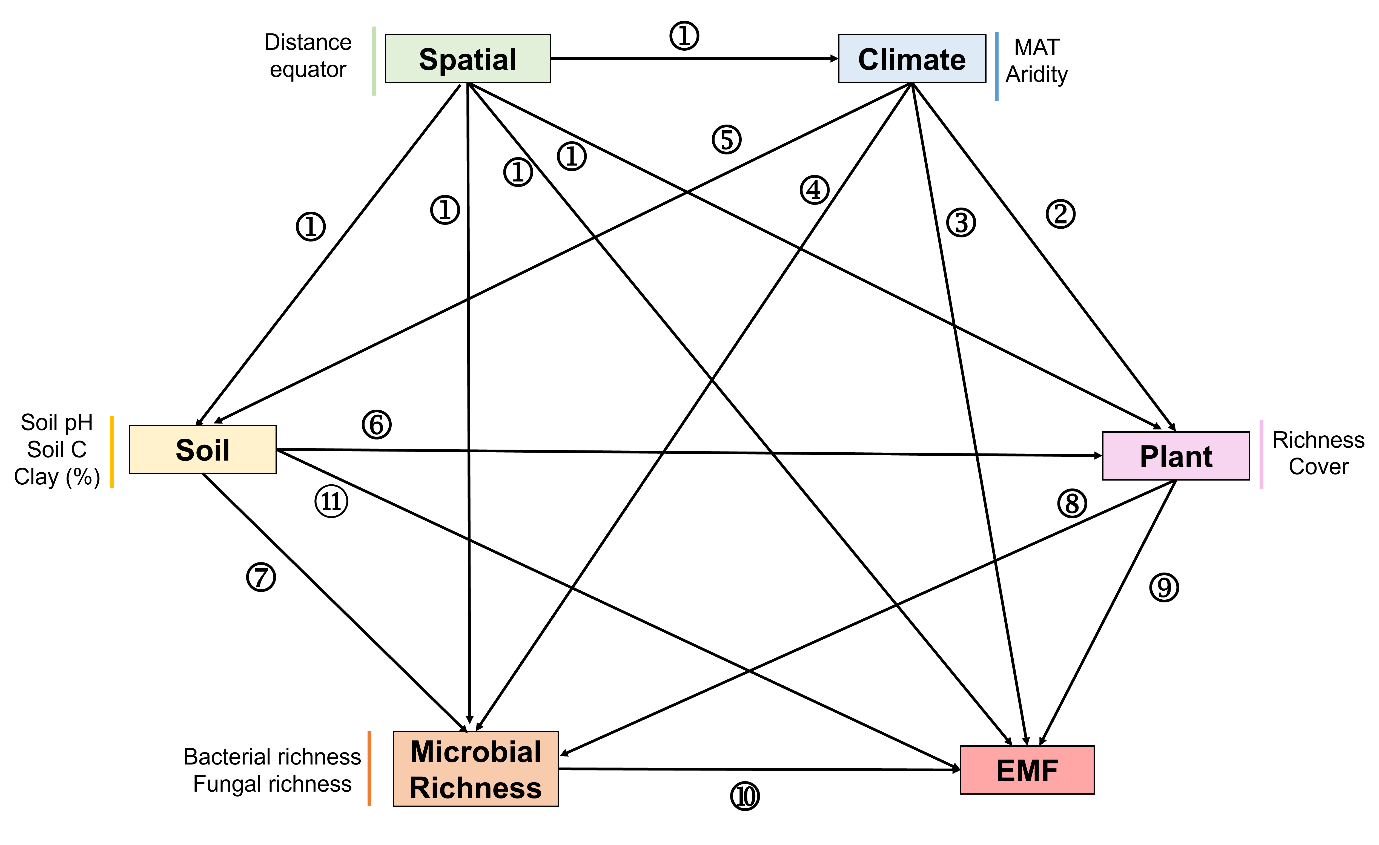


| # | Associations | Rationale | Ref. |
| --- | --- | --- | --- |
| 1 | Spatial → Others | Location is incorporated into our model to account for potential spatial autocorrelation effects. Furthermore, geographic location influences several environmental variables. For instance, as the distance from the equator increases, temperatures tend to decrease. | [1,2] |
| 2 | Climate → Plant attributes | Climate plays a crucial role in regulating plant attributes. For instance, increased aridity is typically associated with a decrease in plant cover and richness. Additionally, on a global scale, mean annual temperature (MAT) tends to be positively correlated with plant cover and richness, as evidenced in tropical regions. | [2,3] |
| 3 | Climate → EMF | Climate is known to control ecosystem multifunctionality. For example, increases in aridity are typically associated with a decrease in ecosystem multifunctionality. | [1,4] |
| 4 | Climate → Microbial richness | Climate controls soil biodiversity on a global scale. For instance, increases in aridity are known to be negatively related to microbial richness. Furthermore, mean annual temperature (MAT) has been reported to be positively associated with bacterial richness. | [5,6] |
| 5 | Climate → Soil attributes | Climate regulates soil properties globally. For example, increases in aridity are negatively associated with soil organic matter (soil C). Mean annual temperature (MAT) is expected to have a positive effect on soil C. Very cold locations, such as alpine ecosystems, are anticipated to have reduced carbon fixation and decomposition rates. Aridity is also expected to increase soil pH by promoting the formation of caliche and altering soil texture by eroding finer fractions, such as clay, through wind erosion. Additionally, aridity can influence clay formation by affecting weathering processes. MAT is expected to affect soil texture and pH by enhancing soil weathering. | [7,8] |
| 6 | Soil → plant attributes | Plant and soil attributes are largely interconnected through various soil-plant processes. For example, plant cover promotes soil carbon (C) through litter production. Additionally, soil fertility enhances plant cover. Soil texture and pH are factors that can control plant cover, but they can also be influenced by plants through weathering and the alteration of soil aggregates. | [9] |
| 7 | Soil → Microbial richness | Soil attributes are among the most important drivers of soil biodiversity. For example, soil pH is a major regulator of bacterial diversity. Soil organic matter is essential for the biodiversity of soil microbes and animals by controlling resource availability. Additionally, soil texture is critical for soil biodiversity as it influences soil aggregates and water availability. | [10,11] |
| 8 | Plant → Microbial richness | Plant attributes are key controllers of microbial richness. For example, plant cover provides resources and habitats for soil organisms. Additionally, plant richness increases the heterogeneity of resources, such as litter heterogeneity, which can enhance the number of niches available for soil organisms. | [12,13] |
| 9 | Plant → EMF | Plant richness and cover are known to positively impact ecosystem multifunctionality. Locations with higher percentages of plant cover can support greater carbon fixation, plant productivity, and resource availability, such as litter production. Additionally, plant richness is expected to enhance ecosystem functioning by promoting resource heterogeneity and facilitating the coexistence of multiple soil organisms. | [14] |
| 10 | Microbial richness → EMF | Belowground organisms comprise a significant portion of global terrestrial biodiversity and are responsible for essential ecosystem functions and services valued at trillions of dollars annually. These functions include plant productivity, nutrient cycling, organic matter decomposition, pollutant degradation, and pathogen control. We hypothesize that soil biodiversity is positively correlated with the rates of multiple ecosystem functions. | [10,15] |
| 11 | Soil → EMF | Soil attributes play a crucial role in controlling soil functioning. Locations with higher amounts of organic matter are expected to support higher rates of organic matter decomposition and nutrient cycling. Soil texture is anticipated to influence ecosystem functioning by regulating water availability and soil aggregation. Finally, pH can significantly affect the rates of ecosystem processes, as enzyme activity and metabolic processes are known to be regulated by pH. | [16] |

# **Figure L.** *A priori* structural equation modeling (SEM) metamodel aimed to evaluate the link between microbial richness and multifunctionality (EMF) after controlling for key ecological predictors such as spatial, climate, and soil and plant attributes. Different categories of predictors were grouped in the model for graphical simplicity. Aridity = - 1 x Aridity Index. MAT = Mean annual temperature. The fitted model is available in Fig 4A.


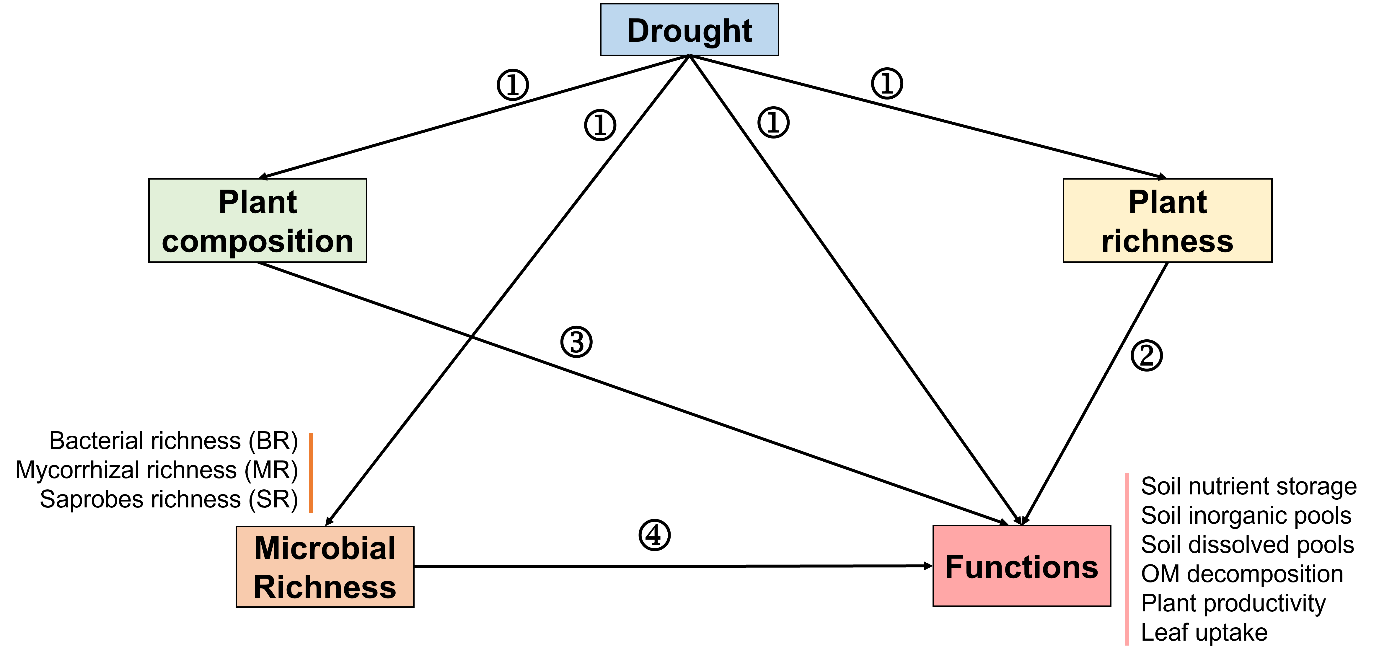


| # | Associations | Rationale | Ref. |
| --- | --- | --- | --- |
| 1 | Drought → Others | Drought can lead to reduced plant growth, decreased productivity, and increased mortality rates. It can reduce soil moisture content, resulting in decreased nutrient availability and altered soil structure. Microbial communities are highly sensitive to drought conditions, which can diminish microbial diversity and activity, thereby affecting crucial processes such as organic matter decomposition and nutrient cycling. | [17] |
| 2 | Plant richness → Functions | Higher plant richness leads to increased biomass production, as diverse plant communities utilize resources more efficiently and occupy different ecological niches. This diversity also promotes more robust nutrient cycling by supporting a variety of decomposer organisms and facilitating different nutrient pathways. Furthermore, ecosystems with higher plant richness tend to be more resilient to environmental disturbances, maintaining functionality and stability under changing conditions. | [18,19] |
| 3 | Plant composition →Functions | Different plant species contribute uniquely to these processes based on their functional traits. For instance, nitrogen-fixing plants can enhance soil fertility, while deep-rooted species improve soil structure and water infiltration. | [20] |
| 4 | Microbial richness →Functions | Microbial richness significantly affects ecosystem functions by enhancing processes such as decomposition, nutrient cycling, and soil formation. A diverse microbial community can perform a wider range of biochemical processes, contributing to more efficient breakdown of organic matter and nutrient availability. | [21] |

# **Figure M.** *A priori* structural equation modeling (SEM) metamodel aimed to evaluate the link between microbial richness and multifunctionality (EMF) after controlling for key ecological predictors such as drought, and plant richness and composition. Different categories of predictors were grouped in the model for graphical simplicity. The fitted model is available in Fig 4B.

# **Table A.** Experimental design describing replicates per diversity and drought treatments for the microcosm study.

| Plant richness | Microbial diversity | Drought | Replicates |
| --- | --- | --- | --- |
| 0 | HD | D | 3 |
| 0 | HD | W | 3 |
| 0 | MD | D | 3 |
| 0 | MD | W | 3 |
| 0 | LD | D | 3 |
| 0 | LD | W | 3 |
| 1 (Rho) | HD | D | 5 |
| 1 (Rho) | HD | W | 3 |
| 1 (Rho) | MD | D | 5 |
| 1 (Rho) | MD | W | 5 |
| 1 (Rho) | LD | D | 4 |
| 1 (Rho) | LD | W | 5 |
| 1 (Dig) | HD | D | 3 |
| 1 (Dig) | HD | W | 3 |
| 1 (Dig) | MD | D | 3 |
| 1 (Dig) | MD | W | 2 |
| 1 (Dig) | LD | D | 3 |
| 1 (Dig) | LD | W | 3 |
| 1 (Rye) | HD | D | 3 |
| 1 (Rye) | HD | W | 3 |
| 1 (Rye) | MD | D | 3 |
| 1 (Rye) | MD | W | 3 |
| 1 (Rye) | LD | D | 3 |
| 1 (Rye) | LD | W | 3 |
| 1 (Pha) | HD | D | 3 |
| 1 (Pha) | HD | W | 2 |
| 1 (Pha) | MD | D | 3 |
| 1 (Pha) | MD | W | 3 |
| 1 (Pha) | LD | D | 3 |
| 1 (Pha) | LD | W | 3 |
| 1 (Luc) | HD | D | 3 |
| 1 (Luc) | HD | W | 3 |
| 1 (Luc) | MD | D | 3 |
| 1 (Luc) | MD | W | 3 |
| 1 (Luc) | LD | D | 3 |
| 1 (Luc) | LD | W | 3 |
| 2 | HD | D | 6 |
| 2 | HD | W | 7 |
| 2 | MD | D | 8 |
| 2 | MD | W | 6 |
| 2 | LD | D | 7 |
| 2 | LD | W | 6 |
| 3 | HD | D | 3 |
| 3 | HD | W | 1 |
| 3 | MD | D | 1 |
| 3 | MD | W | 3 |
| 3 | LD | D | 2 |
| 3 | LD | W | 1 |
| 4 | HD | D | 1 |
| 4 | HD | W | 2 |
| 4 | MD | D | 1 |
| 4 | MD | W | 1 |
| 4 | LD | D | 2 |
| 4 | LD | W | 2 |

# **Table B.** Greenhouse temperature (day/night °C) conditions obtained from monthly averages, of September to January, based on 10-year monthly average data from Meteorological Bureau Station 067021 (<http://www.bom.gov.au>) for the microcosm study.

| Season | Month | Day/night temperature (°C) |
| --- | --- | --- |
| Plant establishment | July | 20/18 |
| Plant establishment | August | 23/18 |
| Spring | September | 23/15 |
| Spring | October | 25/15 |
| Spring | November | 28/18 |
| Summer | December | 29/19 |
| Summer | January | 31/21 |

# **Table C.** Statistical summary (degrees of freedom, F ratio, *p*-values) for the microcosm study, and R^2^ adj of linear mixed model of microbial richness and microbial gene abundance (bacteria: 16S rRNA; fungi: ITS region) at final harvest, when accounting for plant combination variability (n=157). 4-plant richness (1, 2, 3, 4) and 3-plant richness (1, 2, 3) was considered as a co-variate, separately. *P*-values in bold represent significant differences between fixed-effects (*p* < 0.05).

| **Fixed-effects** | **DF** | **4-plant species** | | **3-plant species** | |
| --- | --- | --- | --- | --- | --- |
| **Bacterial richness** |  | **F ratio** | ***p-value*** | **F ratio** | ***p-value*** |
|  |  | **R^2^ adj=0.93** | | **R^2^ adj=0.93** | |
| Plant Richness | 1 | 1.611 | 0.216 | 0.46 | 0.502 |
| Microbial Richness | 2 | 951.250 | **<0.001** | 897.08 | **<0.001** |
| Drought | 1 | 7.728 | **0.006** | 7.95 | **0.006** |
| Plant Richness.Microbial Richness | 2 | 0.202 | 0.817 | 0.73 | 0.483 |
| Plant Richness.Drought | 1 | 0.660 | 0.418 | 0.69 | 0.407 |
| Microbial Richness.Drought | 2 | 0.926 | 0.399 | 1.76 | 0.177 |
| **Fungal richness** |  |  |  |  |  |
|  |  | **R^2^ adj=0.89** | | **R^2^ adj=0.89** | |
| Plant Richness | 1 | 1.539 | 0.237 | 2.76 | 0.122 |
| Microbial Richness | 2 | 527.883 | **<0.001** | 501.99 | **<0.001** |
| Drought | 1 | 1.164 | 0.283 | 2.40 | 0.124 |
| Plant Richness.Microbial Richness | 2 | 0.546 | 0.581 | 1.38 | 0.256 |
| Plant Richness.Drought | 1 | 1.388 | 0.241 | 0.02 | 0.897 |
| Microbial Richness.Drought | 2 | 0.390 | 0.678 | 0.31 | 0.732 |
| **Mycorrhizal fungi richness** |  |  |  |  |  |
|  |  | **R^2^ adj=0.33** | | **R^2^ adj=0.33** | |
| Plant Richness | 1 | 0.349 | 0.564 | 0.107 | 0.749 |
| Microbial Richness | 2 | 26.809 | **<0.001** | 24.832 | **<0.001** |
| Drought | 1 | 1.045 | 0.309 | 0.498 | 0.482 |
| Plant Richness.Microbial Richness | 2 | 0.285 | 0.753 | 0.106 | 0.899 |
| Plant Richness.Drought | 1 | 1.919 | 0.168 | 0.514 | 0.475 |
| Microbial Richness.Drought | 2 | 0.732 | 0.483 | 0.496 | 0.610 |
| **Saprotrophic fungi richness** |  |  |  |  |  |
|  |  | **R^2^ adj=0.89** | | **R^2^ adj=0.89** | |
| Plant Richness | 1 | 2.488 | 0.139 | 3.166 | 0.099 |
| Microbial Richness | 2 | 587.526 | **<0.001** | 543.087 | **<0.001** |
| Drought | 1 | 1.444 | 0.232 | 2.178 | 0.143 |
| Plant Richness.Microbial Richness | 2 | 2.320 | 0.102 | 2.803 | 0.064 |
| Plant Richness.Drought | 1 | 0.314 | 0.576 | 0.035 | 0.852 |
| Microbial Richness.Drought | 2 | 1.103 | 0.335 | 1.074 | 0.345 |
| **Plant pathogens richness** |  |  |  |  |  |
|  |  | **R^2^ adj=0.46** | | **R^2^ adj=0.43** | |
| Plant Richness | 1 | 1.197 | 0.291 | 1.070 | 0.316 |
| Microbial Richness | 2 | 48.303 | **<0.001** | 40.085 | **<0.001** |
| Drought | 1 | 0.770 | 0.382 | 0.183 | 0.669 |
| Plant Richness.Microbial Richness | 2 | 1.207 | 0.302 | 0.221 | 0.802 |
| Plant Richness.Drought | 1 | 1.955 | 0.164 | 0.154 | 0.696 |
| Microbial Richness.Drought | 2 | 0.652 | 0.523 | 0.549 | 0.579 |
| **Bacterial abundance** |  |  |  |  |  |
|  |  | **R^2^ adj=0.28** | | **R^2^ adj=0.26** | |
| Plant Richness | 1 | 6.929 | **0.023** | 16.79 | **0.002** |
| Microbial Richness | 2 | 4.794 | **0.010** | 4.11 | **0.019** |
| Drought | 1 | 2.063 | 0.153 | 1.91 | 0.169 |
| Plant Richness.Microbial Richness | 2 | 0.045 | 0.956 | 0.26 | 0.769 |
| Plant Richness.Drought | 1 | 0.182 | 0.670 | 0.17 | 0.680 |
| Microbial Richness.Drought | 2 | 1.219 | 0.299 | 0.92 | 0.401 |
| **Fungal abundance** |  |  |  |  |  |
|  |  | **R^2^ adj=0.31** | | **R^2^ adj=0.30** | |
| Plant Richness | 1 | 0.046 | 0.833 | 0.00 | 0.984 |
| Microbial Richness | 2 | 1.433 | 0.242 | 0.88 | 0.417 |
| Drought | 1 | 0.091 | 0.763 | 0.06 | 0.814 |
| Plant Richness.Microbial Richness | 2 | 2.777 | 0.066 | 0.04 | 0.964 |
| Plant Richness.Drought | 1 | 0.265 | 0.608 | 0.32 | 0.570 |
| Microbial Richness.Drought | 2 | 0.218 | 0.804 | 0.47 | 0.629 |

# **Table D/1.** Spearman correlation coefficient (ρ) among the 16 functions, and with the weighted multifunctionality index, for the microcosm study. Abbreviations: DOC = dissolved organic C; TDN = total dissolved N; S_C = Soil C; S_N = Soil N; S_P = Soil P; InN = Inorganic N; Phos = Phosphate; Water = Basal respiration; Glucose = Glucose mineralization; Lignin = Lignin degradation; C_cover = Canopy cover; P_height = Plant height; P_biom = Plant biomass; L_C = Leaf C; L_N = Leaf N; L_P = Leaf P; EMF = weighted multifunctionality. Significance is shown in bold (* *p* < 0.05, ** *p* < 0.01 and *** *p* < 0.001).

|  | DOC | TDN | S_C | S_N | S_P | InN | Phos | Water | Glucose | Lignin | C_cover | P_height | P_biom | L_C | L_N | L_P |
| --- | --- | --- | --- | --- | --- | --- | --- | --- | --- | --- | --- | --- | --- | --- | --- | --- |
| DOC |  |  |  |  |  |  |  |  |  |  |  |  |  |  |  |  |
| TDN | **0.80***** |  |  |  |  |  |  |  |  |  |  |  |  |  |  |  |
| S_C | -0.03 | -0.02 |  |  |  |  |  |  |  |  |  |  |  |  |  |  |
| S_N | -0.08 | 0.01 | **0.44***** |  |  |  |  |  |  |  |  |  |  |  |  |  |
| S_P | 0.06 | 0.11 | -0.02 | **0.21**** |  |  |  |  |  |  |  |  |  |  |  |  |
| InN | 0.01 | 0.07 | 0.01 | **0.16*** | 0.09 |  |  |  |  |  |  |  |  |  |  |  |
| Phos | **-0.16*** | -0.15 | 0.03 | **0.21**** | 0.12 | 0.06 |  |  |  |  |  |  |  |  |  |  |
| Water | 0.02 | 0.12 | **0.17*** | 0.09 | -0.01 | -0.02 | -0.08 |  |  |  |  |  |  |  |  |  |
| Glucose | 0.04 | **0.18*** | 0.14 | **0.21**** | 0.15 | 0.05 | 0.10 | **0.31***** |  |  |  |  |  |  |  |  |
| Lignin | 0.05 | 0.09 | 0.05 | -0.08 | -0.05 | -0.02 | -0.03 | **0.31***** | **0.32***** |  |  |  |  |  |  |  |
| C_cover | -0.14 | -0.04 | 0.06 | 0.11 | 0.08 | -0.01 | -**0.20*** | -0.08 | -0.09 | **-0.19*** |  |  |  |  |  |  |
| P_height | -0.14 | -0.08 | -0.03 | 0.13 | 0.10 | **0.19*** | **0.42***** | **-0.16*** | 0.13 | -0.15 | **-0.20*** |  |  |  |  |  |
| P_biom | **-0.25**** | **-0.16*** | 0.07 | 0.13 | 0.09 | -0.05 | **0.25**** | 0.05 | 0.07 | -0.01 | **-0.23**** | **0.44***** |  |  |  |  |
| L_C | 0.04 | -0.02 | -0.03 | -0.05 | -0.10 | 0.14 | 0.09 | -0.08 | -0.05 | -0.13 | 0.10 | **0.51***** | 0.13 |  |  |  |
| L_N | **0.26**** | 0.07 | -0.05 | **-0.21**** | -0.02 | -0.09 | **-0.33***** | -0.02 | -0.11 | -0.12 | 0.01 | **-0.58***** | **-0.59***** | **-0.19*** |  |  |
| L_P | -0.06 | -0.03 | -0.07 | -0.06 | 0.09 | -0.06 | **-0.19*** | -0.10 | 0.01 | 0.06 | -0.14 | **-0.20*** | -0.05 | **-0.30***** | 0.13 |  |
| EMF | **0.41***** | **0.52***** | **0.21**** | **0.37***** | **0.44***** | **0.49***** | **0.16*** | **0.19*** | **0.43***** | **0.22**** | -0.05 | **0.27***** | 0.12 | **0.19*** | -0.11 | 0.12 |

# **Table D/2.** Spearman correlation coefficient (ρ) among ecosystem service categories and weighted multifunctionality for global survey and microcosm study. Significance is shown in bold (* *p* < 0.05, ** *p* < 0.01 and *** *p* < 0.001). Coefficients among individual functions and inorganic pools service and weighted multifunctionality for the global survey were included.

| Microcosm study | | | | | | |
| --- | --- | --- | --- | --- | --- | --- |
|  | Soil dissolved pools | Soil nutrient storage | Soil inorganic pools | OM decomposition | Plant productivity | Leaf uptake |
| Soil dissolved pools |  |  |  |  |  |  |
| Soil nutrient storage | 0.05 |  |  |  |  |  |
| Soil inorganic pools | -0.12 | **0.18*** |  |  |  |  |
| OM decomposition | 0.10 | 0.12 | -0.03 |  |  |  |
| Plant productivity | **-0.24**** | **0.18*** | **0.29***** | -0.09 |  |  |
| Leaf uptake | 0.07 | -0.09 | **-0.18*** | -0.04 | **-0.32***** |  |
| Multifunctionality | **0.47***** | **0.54***** | **0.48***** | **0.35***** | **0.20*** | **0.18*** |

| Global survey | | | | | | |
| --- | --- | --- | --- | --- | --- | --- |
|  | **Plant productivity** | **Nitrate content** | **Ammonium content** | **Available P** | **Inorganic pools** | **Glucose mineralization** |
| Plant productivity |  |  |  |  |  |  |
| Nitrate content | **0.31**** |  |  |  |  |  |
| Ammonium content | **0.53***** | **0.58***** |  |  |  |  |
| Available P | 0.08 | **0.57***** | **0.41***** |  |  |  |
| Inorganic pools | **0.37***** | **0.91***** | **0.78***** | **0.76***** |  |  |
| Glucose mineralization | **0.51***** | **0.44***** | **0.69***** | **0.31**** | **0.57***** |  |
| Weighted Multifunctionality | **0.78***** | **0.61***** | **0.81***** | **0.41***** | **0.73***** | **0.89***** |

# **Table D/3.** Spearman correlation coefficient (ρ) among diversity and composition groups and drought for the microcosm study. Plant composition was obtained from first axis of a PCA and microbial composition from first axis of NMDS. For the global survey, coefficients among diversity and abiotic parameters were also included. Significance is shown in bold (** *p* < 0.01, *** *p* < 0.001).

| Microcosm study | | | | | |
| --- | --- | --- | --- | --- | --- |
|  | Plant richness | Plant composition | Microbial richness | Microbial composition | Plant . Microbial richness |
| Plant richness |  |  |  |  |  |
| Plant composition | -0.05 |  |  |  |  |
| Microbial richness | 0.03 | 0.00 |  |  |  |
| Microbial composition | 0.01 | 0.04 | **-0.91***** |  |  |
| Plant . Microbial richness | **0.63***** | -0.02 | **0.72***** | **-0.65***** |  |
| Drought | 0.00 | -0.01 | -0.08 | 0.03 | -0.05 |
|  |  |  |  |  |  |
| Global survey | | | | | |
|  | Plant richness | Microbial richness | Environment | Aridity Index | Plant . Microbial richness |
| Plant richness |  |  |  |  |  |
| Microbial richness | **0.28**** |  |  |  |  |
| Environment | **0.47***** | 0.12 |  |  |  |
| Aridity Index | **0.59***** | **0.26***** | **0.62***** |  |  |
| Plant . Microbial richness | **0.90***** | **0.64***** | **0.44***** | **0.57***** | - |

# **Table E/1.** *P*-values arising from Spearman correlations in Fig 2A. Significance is shown in bold (*p* < 0.05).

|  | Plant | Bacteria | Fungi | Multitrophic | Mycorrhiza | Saprobes | Plant pathogens |
| --- | --- | --- | --- | --- | --- | --- | --- |
| Multifunctionality | **<0.001** | **0.045** | **<0.001** | **<0.001** | 0.068 | **0.006** | 0.261 |
| Glucose mineralization | **0.003** | 0.197 | **0.005** | **0.001** | **0.036** | 0.171 | 0.912 |
| Ammonium content | **0.044** | 0.963 | 0.079 | **0.023** | 0.055 | 0.563 | 0.526 |
| Available phosphorus | 0.154 | **0.002** | 0.368 | 0.755 | 0.417 | 0.974 | 0.723 |
| Nitrate content | 0.869 | 0.064 | **0.008** | 0.142 | **0.044** | 0.631 | 0.472 |
| Plant productivity | **<0.001** | 0.959 | **<0.001** | **<0.001** | **0.005** | 0.221 | 0.127 |

# **Table E/2.** *P*-values arising from Spearman correlations in Fig 3A. Significance is shown in bold (*p* < 0.05).

|  | Plant | Bacteria | Fungi | Multitrophic | Mycorrhiza | Saprobes | Plant pathogens |
| --- | --- | --- | --- | --- | --- | --- | --- |
| Multifunctionality | **<0.001** | 0.610 | 0.999 | **0.011** | 0.168 | 0.973 | 0.425 |
| Soil nutrient storage | **0.010** | 0.455 | 0.081 | **0.006** | 0.601 | 0.213 | 0.102 |
| Soil inorganic pools | 0.637 | **0.030** | **0.030** | 0.152 | 0.052 | **0.018** | 0.191 |
| Soil dissolved pools | **0.021** | 0.085 | **0.003** | 0.496 | **0.015** | **0.004** | 0.081 |
| OM decomposition | **<0.001** | 0.235 | 0.949 | **0.001** | 0.167 | 0.886 | 0.674 |
| Plant productivity | 0.631 | 0.200 | **0.001** | **0.023** | **0.012** | **0.001** | **0.017** |
| Leaf uptake | 0.883 | 0.473 | **0.040** | 0.223 | **0.023** | **0.028** | 0.623 |
| Soil phosphorus | 0.157 | 0.525 | 0.580 | 0.480 | 0.349 | 0.611 | 0.082 |
| Soil carbon | 0.936 | 0.093 | **0.027** | **0.047** | 0.067 | 0.076 | 0.089 |
| Soil nitrogen | **0.001** | 0.345 | 0.123 | **0.001** | 0.491 | 0.259 | 0.785 |
| Phosphate | 0.806 | 0.501 | 0.496 | 0.532 | 0.285 | 0.265 | 0.619 |
| Inorganic nitrogen | 0.208 | **0.029** | **0.010** | 0.226 | 0.140 | **0.012** | 0.079 |
| Dissolved organic carbon | 0.645 | **0.023** | **0.001** | **0.019** | **0.023** | **0.001** | 0.123 |
| Total dissolved nitrogen | **<0.001** | 0.593 | 0.084 | 0.092 | **0.027** | 0.104 | 0.331 |
| Basal respiration | **<0.001** | **0.029** | 0.331 | **<0.001** | 0.345 | 0.294 | 0.176 |
| Glucose mineralization | **<0.001** | 0.618 | 0.706 | **0.046** | 0.850 | 0.955 | 0.369 |
| Lignin degradation | 0.188 | 0.759 | 0.534 | 0.437 | 0.197 | 0.329 | 0.956 |
| Canopy cover | 0.951 | 0.339 | **0.001** | 0.170 | 0.053 | 0.054 | **0.003** |
| Plant height | **0.018** | 0.102 | 0.355 | 0.673 | 0.060 | **0.036** | 0.957 |
| Plant biomass | 0.192 | 0.894 | 0.328 | 0.138 | 0.346 | 0.126 | 0.525 |
| Leaf carbon | **<0.001** | 0.142 | 0.565 | **0.022** | 0.644 | 0.302 | 0.390 |
| Leaf nitrogen | 0.583 | 0.844 | 0.280 | 0.340 | 0.064 | 0.057 | 0.926 |
| Leaf phosphorus | 0.111 | 0.215 | **0.030** | 0.733 | **0.045** | 0.060 | 0.177 |

# **Table F/1.** *P*-values arising from Variation Partitioning modeling in Fig 2B. Significance is shown in bold (*p* < 0.05).

| Ecosystem property | Plant  richness | Microbial  richness | Environment | Aridity  Index |
| --- | --- | --- | --- | --- |
| Multifunctionality | **<0.001** | **0.002** | **<0.001** | 0.054 |
| Glucose mineralization | 0.112 | **<0.001** | **<0.001** | 0.403 |
| Ammonium content | 0.220 | **0.030** | **<0.001** | 0.934 |
| Nitrate content | 0.174 | **0.015** | **0.001** | 0.695 |
| Available P | 0.486 | **0.037** | **<0.001** | **<0.001** |
| Plant productivity | 0.629 | **0.006** | **<0.001** | 0.019 |

# **Table F/2.** *P*-values arising from Variation Partitioning modeling in Fig 3B. Significance is shown in bold (*p* < 0.05).

| Ecosystem property | Plant  richness | Plant  combination | Microbial  richness | Aridity  Index |
| --- | --- | --- | --- | --- |
| Multifunctionality | **0.001** | **<0.001** | 0.769 | **<0.001** |
| Soil nutrient storage | 0.164 | 0.085 | 0.381 | **<0.001** |
| Soil inorganic pools | 0.769 | 0.013 | 0.137 | 0.547 |
| Soil dissolved pools | **0.044** | 0.115 | **0.040** | 0.558 |
| OM decomposition | **<0.001** | 0.079 | 0.450 | 0.612 |
| Plant productivity | 0.961 | **<0.001** | **0.006** | 0.307 |
| Leaf uptake | 0.361 | **0.007** | 0.123 | 0.224 |
| Soil phosphorus | 0.513 | 0.110 | 0.509 | **<0.001** |
| Soil carbon | 0.965 | 0.759 | 0.079 | 0.150 |
| Soil nitrogen | **<0.001** | 0.052 | 0.311 | **0.001** |
| Phosphate | 0.157 | **<0.001** | 0.612 | 0.270 |
| Inorganic nitrogen | 0.103 | 0.931 | 0.105 | 0.904 |
| Dissolved organic carbon | 0.721 | 0.550 | **0.009** | 0.510 |
| Total dissolved nitrogen | **<0.001** | **0.007** | 0.266 | 0.667 |
| Basal respiration | **<0.001** | 0.775 | 0.089 | 0.510 |
| Glucose mineralization | **<0.001** | **0.001** | 0.766 | 0.746 |
| Lignin degradation | 0.609 | 0.459 | 0.889 | 0.540 |
| Canopy cover | 0.601 | **<0.001** | **0.013** | 0.166 |
| Plant height | **0.015** | **<0.001** | **0.007** | 0.467 |
| Plant biomass | **0.039** | **<0.001** | 0.438 | 0.908 |
| Leaf carbon | 0.077 | 0.134 | 0.890 | 0.616 |
| Leaf nitrogen | **0.028** | **<0.001** | 0.974 | 0.736 |
| Leaf phosphorus | 0.167 | 0.495 | **0.018** | 0.159 |

# **Table G/1.** Statistical summary (degrees of freedom, F ratio, *p*-values) for the microcosm study, and R^2^ adj of linear mixed model of weighted multifunctionality and ecosystem services when accounting for plant combination variability (n=157). 4-plant richness (1, 2, 3, 4) and 3-plant richness (1, 2, 3) was considered as a co-variate. *P*-values in bold represent significant differences between fixed-effects (*p* < 0.05).

| **Fixed-effects** | **DF** | **4-plant species** | | **3-plant species** | |
| --- | --- | --- | --- | --- | --- |
| **Multifunctionality** |  | **F ratio** | ***p-value*** | **F ratio** | ***p-value*** |
|  |  | **R^2^ adj=0.32** | | **R^2^ adj=0.32** | |
| Plant Richness | 1 | 1.810 | 0.199 | 4.13 | 0.058 |
| Microbial Richness | 2 | 1.905 | 0.153 | 1.98 | 0.142 |
| Drought | 1 | 14.164 | **0.000** | 10.99 | **0.001** |
| Plant Richness.Microbial Richness | 2 | 0.593 | 0.554 | 0.74 | 0.481 |
| Plant Richness.Drought | 1 | 1.005 | 0.318 | 0.01 | 0.914 |
| Microbial Richness.Drought | 2 | 0.552 | 0.577 | 0.45 | 0.640 |
| **Soil dissolved pools** |  |  |  |  |  |
|  |  | **R^2^ adj=0.41** | | **R^2^ adj=0.46** | |
| Plant Richness | 1 | 1.340 | 0.268 | 2.09 | 0.172 |
| Microbial Richness | 2 | 2.838 | **0.062** | 3.11 | **0.048** |
| Drought | 1 | 0.343 | 0.559 | 0.27 | 0.604 |
| Plant Richness.Microbial Richness | 2 | 1.226 | 0.297 | 4.39 | **0.014** |
| Plant Richness.Drought | 1 | 0.931 | 0.336 | 1.50 | 0.223 |
| Microbial Richness.Drought | 2 | 0.492 | 0.612 | 0.31 | 0.734 |
| **Soil nutrient storage** |  |  |  |  |  |
|  |  | **R^2^ adj=0.49** | | **R^2^ adj=0.48** | |
| Plant Richness | 1 | 4.944 | **0.043** | 6.38 | **0.021** |
| Microbial Richness | 2 | 14.194 | **<0.001** | 13.81 | **<0.001** |
| Drought | 1 | 78.769 | **<0.001** | 71.60 | **<0.001** |
| Plant Richness.Microbial Richness | 2 | 1.240 | 0.293 | 0.74 | 0.480 |
| Plant Richness.Drought | 1 | 1.425 | 0.235 | 2.31 | 0.131 |
| Microbial Richness.Drought | 2 | 0.271 | 0.763 | 0.32 | 0.729 |
| **Soil inorganic pools** |  |  |  |  |  |
|  |  | **R^2^ adj=0.27** | | **R^2^ adj=0.26** | |
| Plant Richness | 1 | 0.639 | 0.438 | 0.16 | 0.698 |
| Microbial Richness | 2 | 4.758 | **0.010** | 4.36 | **0.015** |
| Drought | 1 | 0.003 | 0.954 | 0.05 | 0.818 |
| Plant Richness.Microbial Richness | 2 | 0.319 | 0.728 | 0.63 | 0.536 |
| Plant Richness.Drought | 1 | 0.816 | 0.368 | 0.20 | 0.653 |
| Microbial Richness.Drought | 2 | 0.532 | 0.589 | 0.69 | 0.505 |
| **OM decomposition** |  |  |  |  |  |
|  |  | **R^2^ adj=0.22** | | **R^2^ adj=0.24** | |
| Plant Richness | 1 | 5.631 | **0.034** | 3.15 | 0.099 |
| Microbial Richness | 2 | 0.659 | 0.519 | 0.56 | 0.570 |
| Drought | 1 | 0.153 | 0.697 | 0.13 | 0.718 |
| Plant Richness.Microbial Richness | 2 | 2.153 | 0.120 | 2.59 | 0.079 |
| Plant Richness.Drought | 1 | 1.552 | 0.215 | 3.07 | 0.082 |
| Microbial Richness.Drought | 2 | 0.121 | 0.886 | 0.11 | 0.897 |
| **Plant productivity** |  |  |  |  |  |
|  |  | **R^2^ adj=0.68** | | **R^2^ adj=0.68** | |
| Plant Richness | 1 | 0.004 | 0.951 | 0.03 | 0.873 |
| Microbial Richness | 2 | 4.012 | **0.020** | 3.62 | **0.030** |
| Drought | 1 | 2.435 | 0.121 | 2.08 | 0.152 |
| Plant Richness.Microbial Richness | 2 | 0.567 | 0.569 | 0.70 | 0.497 |
| Plant Richness.Drought | 1 | 0.058 | 0.810 | 0.01 | 0.927 |
| Microbial Richness.Drought | 2 | 0.284 | 0.753 | 0.33 | 0.716 |
| **Leaf uptake** |  |  |  |  |  |
|  |  | **R^2^ adj=0.19** | | **R^2^ adj=0.16** | |
| Plant Richness | 1 | 0.641 | 0.437 | 0.00 | 0.972 |
| Microbial Richness | 2 | 1.531 | 0.220 | 0.94 | 0.395 |
| Drought | 1 | 1.130 | 0.290 | 0.33 | 0.566 |
| Plant Richness.Microbial Richness | 2 | 0.006 | 0.994 | 0.92 | 0.402 |
| Plant Richness.Drought | 1 | 3.483 | 0.064 | 0.48 | 0.488 |
| Microbial Richness.Drought | 2 | 0.376 | 0.687 | 0.42 | 0.658 |

# **Table G/2.** Statistical summary (degrees of freedom, F ratio, *p*-values) for the microcosm study, and R^2^ adj of linear mixed model of single ecosystem functions when accounting for plant combination variability (n=157). 4-plant richness (1, 2, 3, 4) and 3-plant richness (1, 2, 3) was considered as a co-variate. *P*-values in bold represent significant differences between fixed-effects (*p* < 0.05).

| **Fixed-effects** | **DF** | **4-plant species** | | **3-plant species** | |
| --- | --- | --- | --- | --- | --- |
| **Dissolved Organic C (DOC)** |  | **F ratio** | ***p-value*** | **F ratio** | ***p-value*** |
|  |  | **R^2^ adj=0.36** | | **R^2^ adj=0.42** | |
| Plant Richness | 1 | 0.035 | 0.855 | 0.09 | 0.766 |
| Microbial Richness | 2 | 4.977 | **0.008** | 6.00 | **0.003** |
| Drought | 1 | 0.641 | 0.425 | 0.50 | 0.480 |
| Plant Richness.Microbial Richness | 2 | 0.412 | 0.663 | 3.88 | **0.023** |
| Plant Richness.Drought | 1 | 1.348 | 0.248 | 1.91 | 0.170 |
| Microbial Richness.Drought | 2 | 0.465 | 0.629 | 0.35 | 0.706 |
| **Total Dissolved N (TDN)** |  |  |  |  |  |
|  |  | **R^2^ adj=0.51** | | **R^2^ adj=0.55** | |
| Plant Richness | 1 | 3.925 | 0.069 | 6.62 | **0.023** |
| Microbial Richness | 2 | 0.931 | 0.397 | 0.80 | 0.452 |
| Drought | 1 | 0.068 | 0.794 | 0.06 | 0.801 |
| Plant Richness.Microbial Richness | 2 | 2.844 | 0.062 | 4.54 | **0.012** |
| Plant Richness.Drought | 1 | 0.395 | 0.531 | 0.91 | 0.343 |
| Microbial Richness.Drought | 2 | 0.509 | 0.602 | 0.23 | 0.795 |
| **Soil C** |  |  |  |  |  |
|  |  | **R^2^ adj=0.04** | | **R^2^ adj=0.04** | |
| Plant Richness | 1 | 0.324 | 0.575 | 1.17 | 0.293 |
| Microbial Richness | 2 | 11.266 | **<0.001** | 10.69 | **<0.001** |
| Drought | 1 | 2.878 | 0.092 | 2.75 | 0.100 |
| Plant Richness.Microbial Richness | 2 | 0.444 | 0.643 | 1.31 | 0.274 |
| Plant Richness.Drought | 1 | 0.030 | 0.863 | 0.28 | 0.599 |
| Microbial Richness.Drought | 2 | 0.051 | 0.951 | 0.10 | 0.907 |
| **Soil N** |  |  |  |  |  |
|  |  | **R^2^ adj=0.47** | | **R^2^ adj=0.48** | |
| Plant Richness | 1 | 2.720 | 0.123 | 4.00 | 0.064 |
| Microbial Richness | 2 | 4.924 | **0.009** | 5.57 | **0.005** |
| Drought | 1 | 17.672 | **<0.001** | 18.77 | **<0.001** |
| Plant Richness.Microbial Richness | 2 | 2.126 | 0.123 | 0.61 | 0.547 |
| Plant Richness.Drought | 1 | 11.909 | **0.001** | 10.26 | **0.002** |
| Microbial Richness.Drought | 2 | 0.012 | 0.988 | 0.05 | 0.955 |
| **Soil P** |  |  |  |  |  |
|  |  | **R^2^ adj=0.75** | | **R^2^ adj=0.74** | |
| Plant Richness | 1 | 5.627 | **0.030** | 8.32 | **0.009** |
| Microbial Richness | 2 | 4.351 | **0.015** | 4.15 | **0.018** |
| Drought | 1 | 406.451 | **<0.001** | 362.84 | **<0.001** |
| Plant Richness.Microbial Richness | 2 | 0.287 | 0.751 | 1.40 | 0.250 |
| Plant Richness.Drought | 1 | 4.151 | **0.044** | 2.00 | 0.160 |
| Microbial Richness.Drought | 2 | 0.616 | 0.542 | 0.64 | 0.527 |
| **Inorganic N** |  |  |  |  |  |
|  |  | **R^2^ adj=0.18** | | **R^2^ adj=0.17** | |
| Plant Richness | 1 | 1.021 | 0.330 | 0.16 | 0.695 |
| Microbial Richness | 2 | 3.570 | **0.031** | 3.79 | **0.025** |
| Drought | 1 | 0.233 | 0.630 | 0.25 | 0.617 |
| Plant Richness.Microbial Richness | 2 | 0.059 | 0.943 | 0.64 | 0.527 |
| Plant Richness.Drought | 1 | 0.249 | 0.619 | 0.18 | 0.672 |
| Microbial Richness.Drought | 2 | 0.187 | 0.830 | 0.18 | 0.831 |
| **Phosphate** |  |  |  |  |  |
|  |  | **R^2^ adj=0.23** | | **R^2^ adj=0.24** | |
| Plant Richness | 1 | 0.017 | 0.900 | 0.00 | 0.997 |
| Microbial Richness | 2 | 2.693 | 0.071 | 1.60 | 0.205 |
| Drought | 1 | 1.271 | 0.262 | 0.47 | 0.493 |
| Plant Richness.Microbial Richness | 2 | 0.586 | 0.558 | 0.14 | 0.872 |
| Plant Richness.Drought | 1 | 1.860 | 0.175 | 0.07 | 0.788 |
| Microbial Richness.Drought | 2 | 0.339 | 0.713 | 0.88 | 0.418 |
| **Basal respiration** |  |  |  |  |  |
|  |  | **R^2^ adj=0.26** | | **R^2^ adj=0.25** | |
| Plant Richness | 1 | 4.500 | 0.055 | 1.85 | 0.195 |
| Microbial Richness | 2 | 1.935 | 0.148 | 1.27 | 0.284 |
| Drought | 1 | 0.341 | 0.561 | 0.31 | 0.578 |
| Plant Richness.Microbial Richness | 2 | 2.200 | 0.115 | 2.30 | 0.104 |
| Plant Richness.Drought | 1 | 1.121 | 0.292 | 2.07 | 0.153 |
| Microbial Richness.Drought | 2 | 0.180 | 0.836 | 0.07 | 0.934 |
| **Glucose mineralization** |  |  |  |  |  |
|  |  | **R^2^ adj=0.18** | | **R^2^ adj=0.20** | |
| Plant Richness | 1 | 6.074 | **0.028** | 3.97 | 0.065 |
| Microbial Richness | 2 | 1.828 | 0.165 | 1.91 | 0.152 |
| Drought | 1 | 0.099 | 0.753 | 0.25 | 0.619 |
| Plant Richness.Microbial Richness | 2 | 0.449 | 0.639 | 1.20 | 0.303 |
| Plant Richness.Drought | 1 | 1.076 | 0.301 | 0.67 | 0.414 |
| Microbial Richness.Drought | 2 | 0.092 | 0.912 | 0.24 | 0.787 |
| **Lignin degradation** |  |  |  |  |  |
|  |  | **R^2^ adj=0.13** | | **R^2^ adj=0.16** | |
| Plant Richness | 1 | 0.233 | 0.639 | 0.35 | 0.567 |
| Microbial Richness | 2 | 1.634 | 0.199 | 1.19 | 0.308 |
| Drought | 1 | 0.199 | 0.656 | 0.31 | 0.579 |
| Plant Richness.Microbial Richness | 2 | 1.071 | 0.345 | 2.19 | 0.116 |
| Plant Richness.Drought | 1 | 0.367 | 0.546 | 1.95 | 0.165 |
| Microbial Richness.Drought | 2 | 1.370 | 0.258 | 1.13 | 0.325 |
| **Green canopy cover** |  |  |  |  |  |
|  |  | **R^2^ adj=0.47** | | **R^2^ adj=0.48** | |
| Plant Richness | 1 | 0.028 | 0.871 | 0.02 | 0.880 |
| Microbial Richness | 2 | 4.255 | **0.016** | 3.20 | **0.044** |
| Drought | 1 | 1.820 | 0.180 | 1.61 | 0.207 |
| Plant Richness.Microbial Richness | 2 | 0.727 | 0.485 | 0.34 | 0.712 |
| Plant Richness.Drought | 1 | 0.130 | 0.719 | 0.25 | 0.617 |
| Microbial Richness.Drought | 2 | 0.081 | 0.922 | 0.04 | 0.956 |
| **Plant height** |  |  |  |  |  |
|  |  | **R^2^ adj=0.80** | | **R^2^ adj=0.81** | |
| Plant Richness | 1 | 0.418 | 0.529 | 0.54 | 0.477 |
| Microbial Richness | 2 | 5.828 | **0.004** | 5.26 | **0.006** |
| Drought | 1 | 0.435 | 0.511 | 0.27 | 0.604 |
| Plant Richness.Microbial Richness | 2 | 0.109 | 0.897 | 0.06 | 0.937 |
| Plant Richness.Drought | 1 | 0.019 | 0.891 | 0.38 | 0.539 |
| Microbial Richness.Drought | 2 | 0.614 | 0.543 | 0.54 | 0.586 |
| **Plant biomass** |  |  |  |  |  |
|  |  | **R^2^ adj=0.58** | | **R^2^ adj=0.58** | |
| Plant Richness | 1 | 0.908 | 0.358 | 0.30 | 0.590 |
| Microbial Richness | 2 | 0.142 | 0.868 | 0.08 | 0.926 |
| Drought | 1 | 0.305 | 0.582 | 0.32 | 0.575 |
| Plant Richness.Microbial Richness | 2 | 2.678 | 0.072 | 3.69 | **0.028** |
| Plant Richness.Drought | 1 | 0.695 | 0.406 | 1.11 | 0.295 |
| Microbial Richness.Drought | 2 | 0.374 | 0.689 | 0.40 | 0.673 |
| **Leaf C** |  |  |  |  |  |
|  |  | **R^2^ adj=0.46** | | **R^2^ adj=0.44** | |
| Plant Richness | 1 | 6.717 | **0.022** | 4.72 | **0.047** |
| Microbial Richness | 2 | 1.590 | 0.208 | 1.35 | 0.262 |
| Drought | 1 | 0.537 | 0.465 | 0.68 | 0.413 |
| Plant Richness.Microbial Richness | 2 | 0.400 | 0.671 | 1.07 | 0.346 |
| Plant Richness.Drought | 1 | 0.343 | 0.559 | 0.03 | 0.860 |
| Microbial Richness.Drought | 2 | 0.620 | 0.539 | 0.88 | 0.416 |
| **Leaf N** |  |  |  |  |  |
|  |  | **R^2^ adj=0.70** | | **R^2^ adj=0.71** | |
| Plant Richness | 1 | 0.637 | 0.438 | 0.10 | 0.760 |
| Microbial Richness | 2 | 3.109 | **0.048** | 4.04 | **0.020** |
| Drought | 1 | 0.423 | 0.517 | 0.01 | 0.918 |
| Plant Richness.Microbial Richness | 2 | 1.965 | 0.144 | 1.35 | 0.263 |
| Plant Richness.Drought | 1 | 0.361 | 0.549 | 1.40 | 0.239 |
| Microbial Richness.Drought | 2 | 0.253 | 0.777 | 0.27 | 0.761 |
| **Leaf P** |  |  |  |  |  |
|  |  | **R^2^ adj=0.08** | | **R^2^ adj=0.07** | |
| Plant Richness | 1 | 1.876 | 0.196 | 2.96 | 0.107 |
| Microbial Richness | 2 | 3.308 | **0.040** | 2.74 | 0.068 |
| Drought | 1 | 1.789 | 0.183 | 0.96 | 0.328 |
| Plant Richness.Microbial Richness | 2 | 0.007 | 0.993 | 0.32 | 0.729 |
| Plant Richness.Drought | 1 | 2.794 | 0.097 | 1.09 | 0.298 |
| Microbial Richness.Drought | 2 | 0.453 | 0.637 | 0.50 | 0.606 |

# **Table H.** Partial correlations for the microcosm study, between biodiversity and weighted multifunctionality, controlling for microbial abundance (gene abundance) and community composition (first axis of a NMDS) in the case of microbial richness and plant abundance (total biomass) and plant composition (first axis of a PCA) in the case of plant richness. *P*-values in bold represent significant differences (*p* < 0.05) and underline values are considered marginally significant (*p* < 0.1).

|  | Multifunctionality (controlling for composition) | | Multifunctionality (controlling for abundance) | | Multifunctionality (controlling for composition and abundance) | |
| --- | --- | --- | --- | --- | --- | --- |
| Richness | r | *p*-value | r | *p*-value | r | *p*-value |
| Plant | 0.14 | 0.088 | 0.16 | 0.051 | 0.14 | 0.086 |
|  |  |  |  |  |  |  |
| Bacteria | -0.06 | 0.462 | 0.09 | 0.265 | -0.07 | 0.401 |
|  |  |  |  |  |  |  |
| Fungi | -0.09 | 0.284 | 0.06 | 0.438 | -0.10 | 0.225 |
|  |  |  |  |  |  |  |
| Plant x Microbes | 0.11 | 0.195 | 0.09 | 0.284 | 0.03 | 0.736 |

**References**

1. Maestre, F. T., Quero, J. L., Gotelli, N. J., Escudero, A., Ochoa, V., Delgado-Baquerizo, M., et al. (2012). Plant species richness and ecosystem multifunctionality in global drylands. Science, 335(6065), 214-218. doi:10.1126/science.1215442

2. Parmesan, C., & Yohe, G. (2003). A globally coherent fingerprint of climate change impacts across natural systems. Nature, 421(6918), 37-42.

3. Nguyen, N. H., Song, Z., Bates, S. T., Branco, S., Tedersoo, L., Menke, J., Kennedy, P. G. (2016). FUNGuild: An open annotation tool for parsing fungal community datasets by ecological guild. Fungal Ecology, 20, 241-248. doi:https://doi.org/10.1016/j.funeco.2015.06.006

4. Delgado-Baquerizo, M., Maestre, F. T., Reich, P. B., Jeffries, T. C., Gaitan, J. J., Encinar, D., Singh, B. K. (2016). Microbial diversity drives multifunctionality in terrestrial ecosystems. Nature Communications, 7, 10541. doi:10.1038/ncomms10541

5. Delgado-Baquerizo, M., Oliverio, A.M., Brewer, T.E., Benavent-González, A., Eldridge, D.J., Bardgett, R.D., et al. (2018). A global atlas of the dominant bacteria found in soil. Science, 359(6373), 320-325.

6. Maestre, F.T., Delgado-Baquerizo, M., Jeffries, T.C., Eldridge, D.J., Ochoa, V., Gozalo, B., & et al. (2015). Increasing aridity reduces soil microbial diversity and abundance in global drylands. Proceedings of the National Academy of Sciences, 112(51), 15684-15689.

7. Delgado-Baquerizo, M., Maestre, F.T., Eldridge, D.J., Bowker, M.A., Jeffries, T.C., Singh, B.K., et al. (2013). Biological soil crusts drive key soil processes in global drylands. Nature Communications, 4, 2777.

8. Crowther, T.W., Todd-Brown, K.E.O., Rowe, C.W., Wieder, W.R., Carey, J.C., Machmuller, M.B., et al. (2016). Quantifying global soil carbon losses in response to warming. Nature, 540(7631), 104-108.

9. Cornwell, W.K., Cornelissen, J.H.C., Amatangelo, K., Dorrepaal, E., Eviner, V.T., Godoy, O., et al. (2008). Plant species traits are the predominant control on litter decomposition rates within biomes worldwide. Ecology Letters, 11(10), 1065-1071

10. Bardgett, R. D., & van der Putten, W. H. (2014). Belowground biodiversity and ecosystem functioning. Nature, 515(7528), 505-511. doi:10.1038/nature13855.

11. Fierer, N., & Jackson, R.B. (2006). The diversity and biogeography of soil bacterial communities. Proceedings of the National Academy of Sciences, 103(3), 626-631.

12. Hooper, D. U., Bignell, D. E., Brown, V. K., Brussard, L., Dangerfield, J. M., Wall, D. H., et al. (2000). Interactions between Aboveground and Belowground Biodiversity in Terrestrial Ecosystems: Patterns, Mechanisms, and Feedbacks: We assess the evidence for correlation between aboveground and belowground diversity and conclude that a variety of mechanisms could lead to positive, negative, or no relationship—depending on the strength and type of interactions among species. BioScience, 50(12), 1049-1061. doi:10.1641/0006-3568(2000)050[1049:Ibaabb]2.0.Co;2

13. Wardle, D. A., Bardgett, R. D., Klironomos, J. N., Setälä, H., van der Putten, W. H., & Wall, D. H. (2004). Ecological Linkages Between Aboveground and Belowground Biota. Science, 304(5677), 1629-1633. doi:10.1126/science.1094875

14. Lefcheck, J.S., Byrnes, J.E.K., Isbell, F., Gamfeldt, L., Griffin, J.N., Eisenhauer, N., et al. (2015). Biodiversity enhances ecosystem multifunctionality across trophic levels and habitats. Nature Communications, 6, 6936.

15. Wagg, C., Bender, S. F., Widmer, F., & van der Heijden, M. G. A. (2014). Soil biodiversity and soil community composition determine ecosystem multifunctionality. Proceedings of the National Academy of Sciences, 111(14), 5266-5270. doi:10.1073/pnas.1320054111

16. Rousk, J., Brookes, P.C., & Bååth, E. (2009). Contrasting soil pH effects on fungal and bacterial growth suggest functional redundancy in carbon mineralization. Applied and Environmental Microbiology, 75(6), 1589-1596.

17. Schimel, J., Balser, T.C., & Wallenstein, M. (2007). Microbial stress-response physiology and its implications for ecosystem function. Ecology, 88(6), 1386-1394.

18. Isbell, F., Calcagno, V., Hector, A., Connolly, J., Harpole, W. S., Reich, P. B., et al. (2011). High plant diversity is needed to maintain ecosystem services. Nature, 477(7363), 199-202. doi:10.1038/nature10282

19. Tilman, D., Reich, P.B., & Knops, J.M.H. (2006). Biodiversity and ecosystem stability in a decade-long grassland experiment. Nature, 441(7093), 629-632

20. Diaz, S., & Cabido, M. (2001). Vive la différence: Plant functional diversity matters to ecosystem processes. Trends in Ecology & Evolution, 16(11), 646-655.

21. Van Der Heijden, M. G. A., Bardgett, R. D., & Van Straalen, N. M. (2008). The unseen majority: soil microbes as drivers of plant diversity and productivity in terrestrial ecosystems. Ecology Letters, 11(3), 296-310. doi:10.1111/j.1461-0248.2007.01139.x
